# An anti-inflammatory and neuroprotective biomimetic nanoplatform for repairing spinal cord injury

**DOI:** 10.1016/j.bioactmat.2022.05.026

**Published:** 2022-06-02

**Authors:** Xiang Gao, Zhihui Han, Cheng Huang, Huali Lei, Guangqiang Li, Lin Chen, Dandan Feng, Zijie Zhou, Qin Shi, Liang Cheng, Xiaozhong Zhou

**Affiliations:** a Department of Orthopedics, The Second Affiliated Hospital of Soochow University, Suzhou, Jiangsu, 215004, China; b Institute of Functional Nano & Soft Materials (FUNSOM), Collaborative Innovation Center of Suzhou Nano Science and Technology, Soochow University, Suzhou, Jiangsu, 215123, China; c Department of Orthopedics, The First Affiliated Hospital of Soochow University, Orthopedic Institute, Soochow University, 188 Shizi Road, 215006, Suzhou, Jiangsu, China

**Keywords:** Biomimetic nanomaterials, ROS scavenging, Anti-inflammatory, Spinal cord injury, Neuro-protective

## Abstract

Spinal cord regeneration after a spinal cord injury (SCI) remains a difficult challenge due to the complicated inflammatory microenvironment and neuronal damage at the injury sites. In this study, retinoic acid (RA) and curcumin (Cur) were co-loaded with bovine serum albumin (BSA) self-assembly to obtain RA@BSA@Cur nanoparticles (NPs) for SCI treatment. Cur, as an antioxidant drug, facilitated reactive oxygen species (ROS) scavenging, and decreased the amount of inflammatory factors secreted by macrophages, while RA could enhance neurite extensions and neural differentiation. The constructed RA@BSA@Cur NPs not only induced polarization of macrophages toward pro-regenerative phenotypes and markedly reduced the inflammatory response of macrophages or microglia, but also increased neurite length in PC12 cells and neuronal differentiation of bone marrow mesenchymal stem cells, improved the differentiation of neural stem cells (NSCs) into β3-tubulin^+^ neurons, and reversed the pro-astrocyte differentiation effect of inflammatory cytokines on NSCs. *In vivo* experiments revealed that RA@BSA@Cur NPs regulated the phenotypic polarization of macrophages, inhibited the release of inflammatory mediators, promoted functional neuron regeneration and motor function, and further inhibited scar tissue formation. This study highlighted that the BSA-based biomimetic nanomaterials could be used as ROS scavengers and nerve regeneration promoters for treating SCI.

## Highlights

• Retinoic acid and Curcumin with remarkable neuroprotective and antioxidant activities are prepared for spinal cord injury.
• RA@BSA@Cur NPs induce polarization of macrophages, reduce levels of tumor necrosis factor-α and interleukin-6, and thus reduce the inflammatory response.
• RA@BSA@Cur NPs increase neurite length in PC12 cells and neuronal differentiation of bone marrow mesenchymal stem cells, and regulate the neurons/astrocytes differentiation of NSCs.
• RA@BSA@Cur NPs reverse the pro-astrocyte differentiation effect of macrophages-derived inflammatory cytokines on NSCs.
• RA@BSA@Cur NPs promote neuron regeneration and motor function, inhibit scar tissue formation, and regulate the phenotypic polarization of macrophages.

## Graphical abstract

**Figure:**
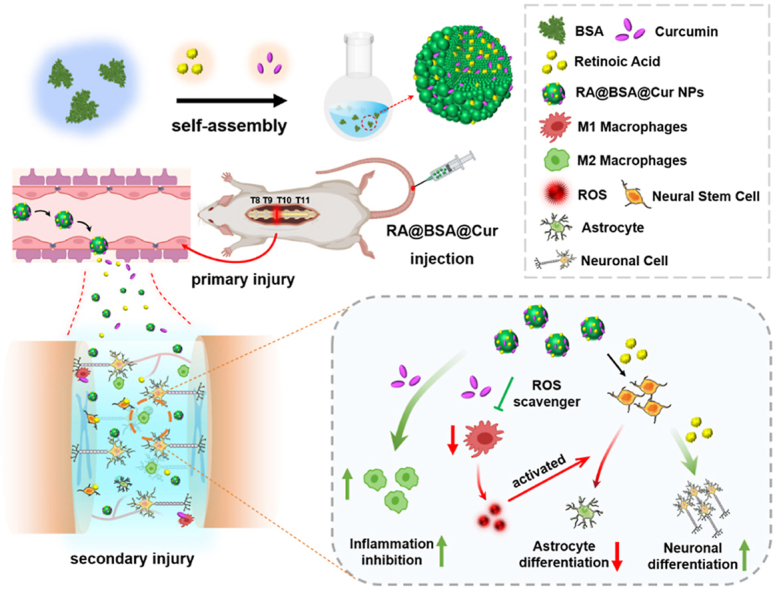

## Teaser

*The BMSCs Differentiation with RA@BSA@Cur NPs:* Mice BMSCs were supplied by Wuhan Procell Life Science & Technology Co.,Ltd. The cells (2.0 × 10^4^ cells/mL) were cultured in 24-well plate with DMEM medium (low glucose) containing 10% FBS, treated with different BSA nanoparticles for 7 days. The cells were stained with anti-β3-tubulin antibody (Abcam, ab78078, USA), and anti-MAP2 antibody (Affinity Biosciences, AF4081, USA) respectively. The primary antibody was marked with secondary antibody (Alexa647, Abcam, ab150083, USA). The F-actin protein was stained with FITC-phalloidin. The cell nucleus was stained with DAPI.

## 1 Introduction

Every year, half a million people suffer from traumatic spinal cord injury (SCI) in global, leading to motor or sensory function impairment [1,2]. However, the clinical treatments for SCI patients are limited because of neuronal apoptosis, infiltration of glial cells and macrophages at the injury site, inflammation after the primary insult, and complex secondary injury cascade in central nervous system (CNS) [[3], [4], [5]]. This cascade is followed by injury to blood–spinal cord barrier and inflammatory cell infiltration; a glial cells-derived protective barrier quickly forms to resist the damage propagation [6,7]. The natural defenses by the barrier cause endogenous inflammatory reactions; therefore, they stimulate secondary responses such as generation of reactive oxygen species (ROS), axonal demyelination, and neuronal cell necrosis [8,9]. Multiple factors of damage increase neural stem cells (NSCs) accompanied with the stem cell differentiation to initiate neural protection and repair. The inhospitable microenvironment at the injury area supports astrocytic differentiation and hinders neuronal regeneration [10]. Therefore, reducing the levels of inflammatory factors and re-establishing a microenvironment conducive for neuronal regeneration is a promising strategy for SCI treatment.

In the early stages of SCI, blood-borne immune cells infiltrate and differentiate into macrophages, which constitute innate immunity along with microglia [11]. The inflammatory reaction by macrophages is the characteristic of neuro-inflammation, which could be effected by lipopolysaccharide (LPS) and ROS [12,13]. Macrophages exhibit two types of effects in neuro-inflammation, which are attributed to the classically activated type 1 (M1) or alternatively activated type 2 (M2) macrophages [14]. M1 macrophages kill neighboring cells and inhibit cellular proliferation, whereas M2 macrophages promote cell activation and tissue growth [15]. Hence, regulating the response of macrophages may contribute to controlling inflammatory response in SCI. Curcumin (Cur), as a prototype natural product, is an antioxidant and anti-inflammatory agent [16]. Cur reduces infiltration of inflammatory macrophages and polarizes M0/M1 macrophages to M2 phenotype in SCI [17]. In addition, Cur promotes the proliferation, survival, and invasion of NSCs [18]. NSCs can differentiate into neurons, astrocytes, and oligodendrocytes in the spinal cord. However, efficiency of the NSC-derived differentiation to neurons is not high due to limited anti-inflammatory effect. Considering the effect of Cur, the application of this pro-differentiation agent can further enhance NSC-derived production of neurons and promote recovery of spinal cord function. Retinoic acid (RA), as a therapeutic agent, induced axon regeneration and NSC-derived differentiation to neurons [19]. However, the inflammatory cytokines reduce the neurotropic effect of RA in NSC differentiation. Although Cur and RA may complement each other in SCI, they have various drawbacks such as degradation at physiological conditions and poor water solubility.

**Scheme 1:**
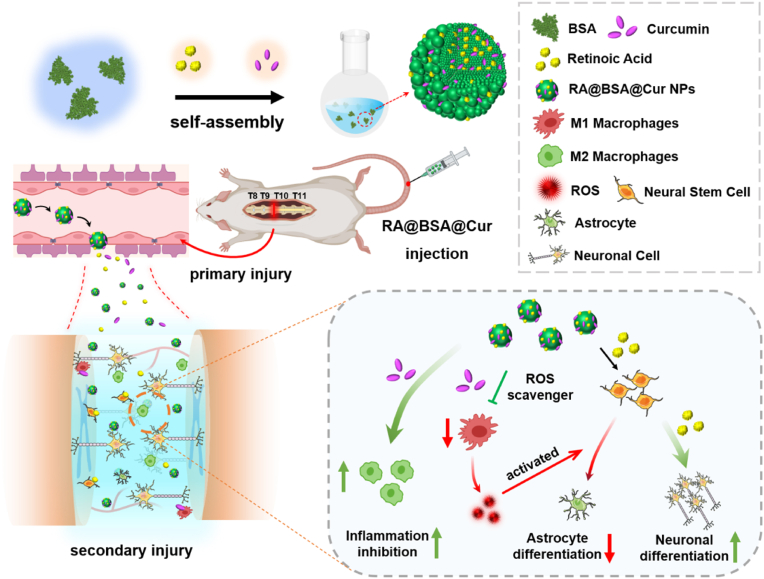
**Schematic illustration of the RA@BSA@Cur NPs for the complete transection spinal cord repair**. Retinoic acid (RA) and curcumin (Cur) were co-loaded inside bovine serum albumin (BSA) via self-assembly method and formed RA@BSA@Cur NPs for the treatment of spinal cord injury (SCI).

Albumin, an FDA-approved protein, has been widely used in drug delivery systems because of its properties including easy drug loading, good water solubility, and nontoxicity [20]. In addition, albumin can bind to the albumin receptor and transfer the drug into the extravascular space via caveolae formation for targeted delivery of drugs [21]. In this study, a novel nanodrug delivery system with RA and Cur (RA@BSA@Cur NPs) was established using bovine serum albumin (BSA). RA@BSA@Cur NPs were optimized to scavenge ROS, regulate macrophage polarization, and regulate neuronal/astroglial differentiation of NSCs (Scheme 1). RA@BSA@Cur NPs could balance M1/M2 polarization ratio and reduce tumor necrosis factor-α (TNF-α) and interleukin-6 (IL-6). RA@BSA@Cur NPs increased neurite length and neuronal differentiation of bone marrow mesenchyml stem cells (BMSCs) *in vitro* in PC12 cells. The RA@BSA@Cur NPs not only induced differentiation of NSCs into β3-tubulin^+^ neurons but also reduced NSC-derived astrocytes production by regulating M1 polarization. In addition, *in vivo* experiments demonstrated that RA@BSA@Cur NPs could correct the macrophage imbalance, inhibit the release of inflammatory factors, promote the production of neurons and axons, and hinder scar tissue hyperplasia. Our study indicated that RA@BSA@Cur NPs could be used as anti-inflammatory and pro-differentiation agent for treating certain diseases of CNS including SCI.

## 2 Results and discussion

### 2.1 Preparation and characterization of RA@BSA@Cur NPs

**Fig. 1:**
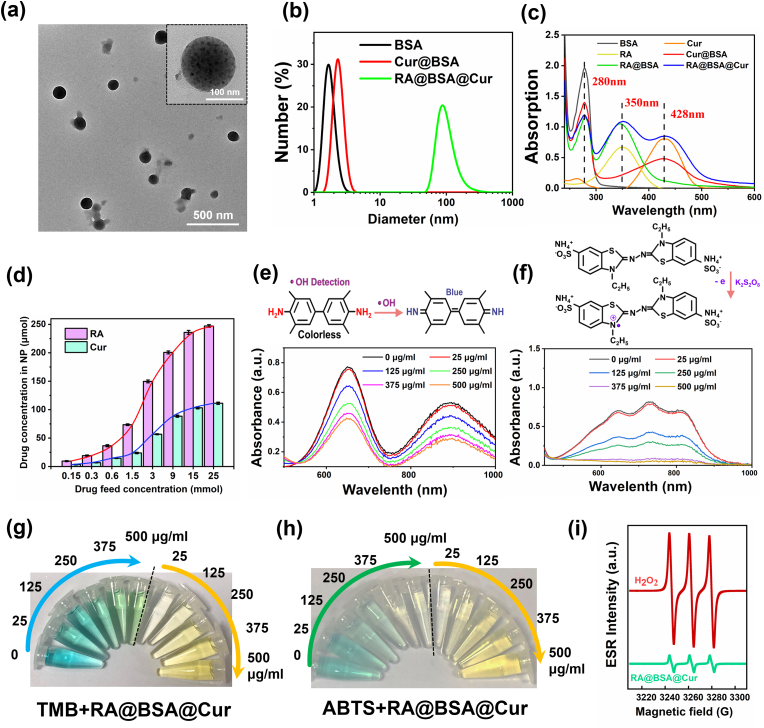
**Characterization of RA@BSA@Cur NPs.** (**a**) TEM images of RA@BSA@Cur NPs with low and high magnification. (**b**) DLS data of different BSA NPs in solutions. (**c**) UV–vis spectra of BSA, RA, Cur, RA@BSA, Cur@BSA and RA@BSA@Cur NPs. (**d**) The loading concentration of RA and Cur in BSA. (**e**) The concentration-dependent anti-oxidation of TMB due to •OH elimination by RA@BSA@Cur NPs with 100 μM H_2_O_2_. (**f**) The concentration-dependent investigation of ABTS^•+^ in the presence of RA@BSA@Cur NPs. (**g**) The change in color of TMB treated with different RA@BSA@Cur NPs. (**h**) The change in color of ABTS^•+^ treated with different RA@BSA@Cur NPs. (**i**) ROS scavenging activities of RA@BSA@Cur NPs for ^1^O_2_ studied by the ESR spectroscopy.

BSA biomimetic nanoparticles carrying RA and Cur and having anti-inflammatory and pro-differentiation action were synthesized via self-assembly method. In brief, BSA was loaded with RA at the molar ratio of 3:1 (RA:BSA) by hydrophilic and hydrophobic interactions [22], forming RA-labeled BSA NPs. Further, Cur, as a typical anti-inflammatory drug, was loaded onto RA@BSA NPs at the molar ratio of 3:1 (Cur:BSA) via the same hydrophilic and hydrophobic interactions to form RA@BSA@Cur NPs. The size of RA@BSA@Cur NPs could be changed by changing the ratio of RA and Cur. Transmission electron microscopy revealed the uniform morphology of RA@BSA@Cur NPs with the size approximately 90 nm (Fig. 1a). Dynamic light scattering (DLS) revealed that the hydrodynamic sizes changed from approximately 4-90 nm after the self-assembly of Cur@BSA with RA (Fig. 1b). UV-vis-NIR revealed that absorption spectra of RA@BSA@Cur NPs contained absorption peak of RA (300–400 nm) and Cur (400–500 nm) (Fig. 1c). After the detailed calculation, BSA exhibited excellent drug loading ability, and the drug loading concentrations were approximately 240 μM for RA and approximately 100 μM for Cur at pH 7.4 (Fig. 1d). In addition, RA@BSA@Cur NPs exhibited an excellent stability in ultrapure water (Figs. S1–S2, Supporting Information). Importantly, to simulate the stability of NPs in physiological solutions, we assessed the change in sizes of RA@BSA@Cur NPs in phosphate buffered saline (PBS) and DMEM solutions at various pH. DLS revealed that the size of RA@BSA@Cur NPs was still approximately 100 nm in both the solutions (Fig. S3a, Supporting Information). In addition, the sizes of RA@BSA@Cur NPs in PBS at various pH were still approximately 90–150 nm, without any obvious change within 3 days (Figs. S3b–d, Supporting Information). The hemolytic test revealed minimal toxicity of RA@BSA@Cur NPs in erythrocytes (Fig. S4, Supporting Information).

### 2.2 ROS scavenging performance of RA@BSA@Cur NPs

The anti-inflammatory action of RA@BSA@Cur NPs was carefully investigated. 3,3,5,5-tetramethylbenzidine (TMB), a commonly used ROS probe, was used for quantitative analysis of hydroxyl radical (•OH) elimination by RA@BSA@Cur NPs. The characteristic absorption peak of TMB at approximately 652 nm was gradually decreased, indicating that •OH was eliminated by the Cur from RA@BSA@Cur NPs (Fig. 1e and g). Using 2,2′-Azinobis-(3-ethylbenzthiazoline-6-sulphonate) (ABTS), the antioxidant properties and scavenging efficiency of ABTS^•+^ were measured. With the increasing concentrations of ABTS, the antioxidant activity of RA@BSA@Cur NPs gradually increased (Fig. 1f and h). Electron spin resonance was used to monitor singlet dioxygen (^1^O_2_) scavenging ability and further prove the ROS scavenging ability of RA@BSA@Cur NPs. The characteristic peak of free radical of 2,2,6,6-tetramethylpiperidine 1-oxyl (1: 1: 1) remarkably reduced after treating with RA@BSA@Cur NPs (Fig. 1i), indicating the singlet oxygen was efficiently eliminated by the NPs. The ROS inhibition ratios by RA@BSA@Cur NPs were calculated to be approximately 96.53%, 46.75%, and 88.73% for ABTS^•+^, •OH, and ^1^O_2_, respectively, at their highest concentration (Fig. S5, Supporting Information). This demonstrated that RA@BSA@Cur NPs exhibited a good ROS scavenging ability, and it can act as a good antioxidant.

### 2.3 RA@BSA@Cur NPs regulated M1/M2 macrophage polarization

**Fig. 2:**
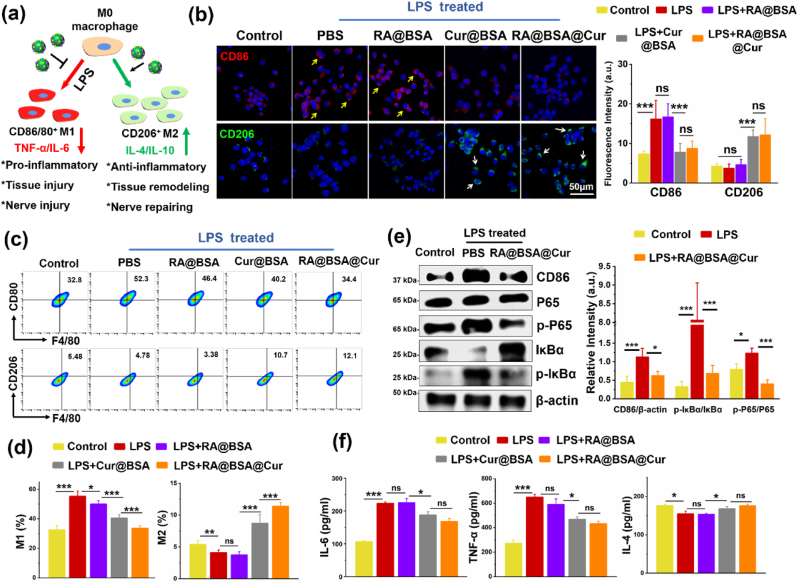
**Macrophage phenotype regulation based on RA@BSA@Cur NPs.** (**a**) The schematic illustration of RA@BSA@Cur NPs regulated macrophage polarization under LPS. (**b**) The immunofluorescence and quantification results of RAW264.7 cultured different BSA-related NPs in LPS condition. CD86^+^ M1 macrophage (yellow arrow) and CD206^+^ M2 macrophage (white arrow), nuclei (DAPI: blue). Scale bar, 50 μm. (**c**&**d**) The flow cytometry analysis and quantification results of RAW264.7 (gated on F4/80^+^) cultured different BSA-related NPs in LPS condition. (**e**) The macrophages' M1 inhibition and M2 promotion regulated by RA@BSA@Cur NPs may be through the NF-*κ*B pathway. (**f**) The inflammatory factors IL-6, TNF-α, and IL-4 changes after RA@BSA@Cur treatment. (n = 3 independent samples). Statistical differences were determined by using the Analysis of Variance (ANOVA) with Bonferroni's multiple comparison test (*p < 0.05, **p < 0.01, ***p < 0.001, ns: no significant; a.u. means arbitrary unit).

The immune response of macrophages with the treatment of RA@BSA@Cur NPs was studied (Fig. 2a). LPS-treated macrophages exhibited M1 phenotype (CD86^high^), and RA@BSA@Cur NPs inhibited M1-like polarization (Fig. 2b). Similarly, the macrophages expressed CD86^low^ fluorescence in Cur@BSA group, indicating that Cur prevented the macrophages from polarizing toward the M1 type. The flow cytometric analysis revealed that the levels of CD80^+^/F4/80^+^ macrophages lowered after co-incubation with RA@BSA@Cur NPs for 24 h, whereas those of CD206^+^/F4/80^+^ macrophages slightly increased (Fig. 2c–d). A similar observation was noted in Cur@BSA group. However, the M1 polarization ratio in RA@BSA NPs or PBS + LPS group reached 50%–60%, further indicating that RA@BSA@Cur NPs could inhibit the inflammatory response that inhibited M1 polarization and could increase M2-polarization. Western blotting revealed that the CD86 levels in the LPS-treated group were higher than those in the control group. This indicated that macrophages induced by LPS were in the inflammatory state, and CD86 levels were increased. When RA@BSA@Cur NPs were used, the levels of CD86 were notably decreased. Compared with the control group, LPS significantly increased the phosphorylated-I*κ*B*α* (p-IκBα) and p-p65 expression. However, this effect was partially blocked by RA@BSA@Cur NPs (Fig. 2e). The total I*κ*B*α* levels in the LPS group were lower than those in the RA@BSA@Cur group. The levels of P65 proteins were not reduced. These results demonstrated that RA@BSA@Cur NPs inhibited M1 macrophages and promoted M2 macrophages probably through the nuclear factor kappa B (NF-*κ*B) pathway. Further, the inflammatory cytokines of the macrophages treated with RA@BSA@Cur NPs were examined. The levels of proinflammatory cytokines, such as IL-6 and TNF-α, were decreased and those of anti-inflammatory factor IL-4 were slightly promoted in both the RA@BSA@Cur and Cur@BSA groups (Fig. 2f) compared with the PBS + LPS groups. To confirm the antioxidative effect of RA@BSA@Cur NPs in immune cells, the ROS probe DCFH-DA was used. The intracellular ROS levels in BV2 microglial cells were assessed as per the DCFH-DA fluorescence intensity. The PBS + LPS group exhibited a high DCFH-DA signal, indicating that the inflammatory mediators induced ROS in the microglial cells and triggered a strong inflammatory response. However, in the RA@BSA@Cur and Cur@BSA groups, low levels of ROS were generated with LPS stimulation. This demonstrated that Cur-loaded BSA NPs could inhibit ROS generated in neuro-immune cells (Fig. S6, Supporting Information). Collectively, these results demonstrated that RA@BSA@Cur NPs with ROS scavenging activity could inhibit polarization to M1 macrophages, promoting anti-inflammatory cytokines secretion in immune cells *in vitro*.

### 2.4 RA@BSA@Cur NPs effected the proliferation and neurite outgrowth

**Fig. 3:**
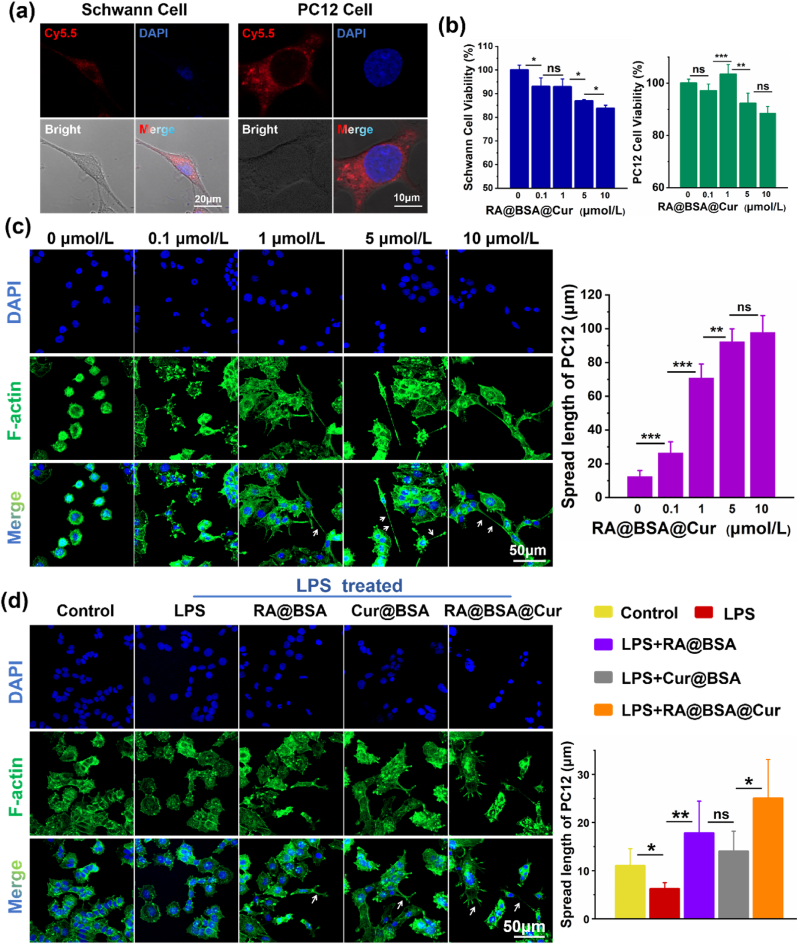
**RA@BSA@Cur NPs effected the neurite outgrowth of PC12 cell**. (**a**) Fluorescence images of Schwann cells and PC12 cells incubated with Cy5.5-labeled RA@BSA@Cur NPs for 6 h. Scale bar, 10 μm. (**b**) The relative viabilities of Schwann cell and PC12 cell incubated with different concentrations of RA@BSA@Cur NPs for 24 h. (**c**) The neurite length (white arrow) of PC12 cells cultured with different RA@BSA@Cur concentrations. Scale bar, 50 μm. (**d**) The neurite length of PC12 cells cultured with RA@BSA, Cur@BSA, and RA@BSA@Cur NPs in pathological condition. Scale bars, 50 μm. (n = 3 independent samples). Statistical differences were determined by using the ANOVA with Bonferroni's multiple comparison test (*p < 0.05, **p < 0.01, ***p < 0.001, ns: no significant; a.u. means arbitrary unit).

The Schwann cells (SCs) and PC12 cells were used to assess the cytocompatibility and neurite outgrowth function. The efficiency of SCs and PC12 cells to endocytose Cy5.5-labeled RA@BSA@Cur NPs was studied. Significant level of red fluorescence was observed in their cell cytoplasm after 6 h incubation with the NPs, indicating that the NPs were efficiently endocytosed into cell cytoplasm (Fig. 3a). We measured the effect of RA@BSA@Cur NPs on cell viability using cell counting kit-8 (CCK-8) assay. SCs and PC12 cells were incubated with various concentrations of RA@BSA@Cur NPs for 24 h. RA@BSA@Cur NPs promoted cell proliferation at a relatively low concentration (Fig. 3b). The reasons could be as follows: (1) It has been reported that small doses of Cur promoted SCs proliferation through runt-related transcription factor 2 (Runx2). However, high concentrations of Cur exhibited no obvious pro-proliferation effect [23]. (2) Low concentrations of RA stimulated proliferation of PC12 cells.However, no increase in cell proliferation was observed when higher concentrations of RA were used [24]. Further, PC12 cells were treated with various concentrations of RA@BSA@Cur NPs. The long neurite outgrowth appeared at 1, 5, and 10 μmol/L of RA@BSA@Cur NPs, where the neurite length of PC12 cells was approximately 70–100 μm. No obvious neurites appeared at 0–0.1 μmol/L of RA@BSA@Cur NPs. Considering the cell proliferation results, we selected 1 μmol/L as the optimum concentration of RA@BSA@Cur NPs for further experiments (Fig. 3c). To further compare the differences among the BSA NPs groups in terms of neurite outgrowth, the low-differentiated PC12 cells were incubated with RA@BSA, Cur@BSA, and RA@BSA@Cur NPs. The changes in neurite outgrowth were observed. Compared with the control and Cur@BSA groups, the neurite length in PC12 cells was approximately 20 μm in the control and Cur@BSA groups. However, the neurite length expanded to approximately 115 μm in the RA@BSA and RA@BSA@Cur groups (Fig. S7, Supporting Information). At pathological conditions, LPS inhibited neurite elongation in PC12 cells (approximately 5 μm). Although neurite has no obvious elongation in the pathological conditions, the neurite length in the RA@BSA (approximately 19 μm) and RA@BSA@Cur (approximately 27 μm) groups was still higher than that in the other groups (Fig. 3d). Collectively, these results confirmed the action of RA@BSA and RA@BSA@Cur NPs on the neurite outgrowth. Therefore, RA@BSA@Cur NPs might establish suitable niche to stimulate the proliferation and neurite outgrowth of neural cells.

### 2.5 RA@BSA@Cur NPs promoted neuronal differentiation of BMSCs

The *trans*-differentiation detection of BMSCs into neurons provides a novel evaluation system for neurofunctional materials [25]. The morphology of BMSCs at passage 3 was spindle-shaped. The induction medium was prepared using low-glucose DMEM with 10% fetal calf serum, including one of RA@BSA, Cur@BSA, or RA@BSA@Cur NPs. The BMSCs were incubated in the induction medium for 7 days. It was observed that the morphology of cells in the RA@BSA and RA@BSA@Cur groups was changed, and they resembled neurons. Compared with the control and Cur@BSA groups, the differentiated cells in the RA@BSA and RA@BSA@Cur groups were positive for β3-tubulin and MAP2 and appeared hyperchromatic. Immunocytochemical staining revealed that RA@BSA and RA@BSA@Cur NPs promoted differentiation of BMSCs into neurons, as demonstrated by the increased fluorescence intensity of β3-tubulin in RA@BSA@Cur NPs (21 ± 5.6) compared with that in the control group (3.2 ± 0.4; p < 0.05) (Fig. S8, Supporting Information). Similarly, the percentage of MAP2^+^/F-actin^+^ cells increased after the treatment with RA@BSA and RA@BSA@Cur NPs compared with the control group (p < 0.05; Fig. S9, Supporting Information). The immunocytochemical staining results indicated that pretreatment of RA@BSA@Cur NPs may lead to *trans*-differentiation of BMSCs into mature neuron-like cells.

### 2.6 RA@BSA@Cur NPs regulated the NSCs differentiation in pathological condition

**Fig. 4:**
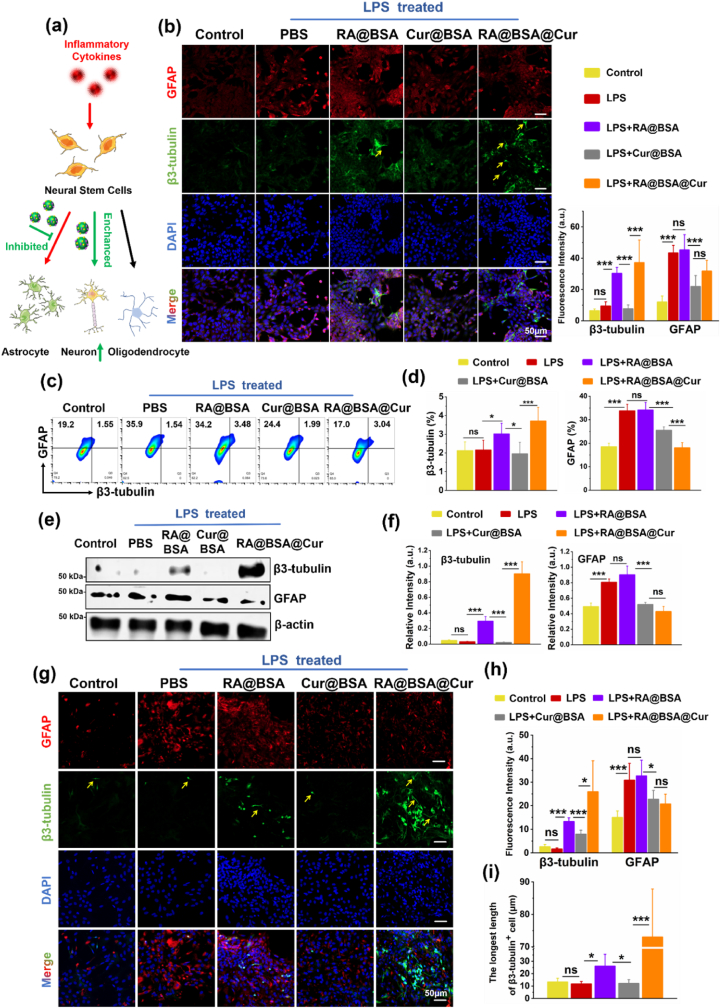
**RA@BSA@Cur NPs effected the neural stem cells differentiation.** (**a**) The schematic illustration of RA@BSA@Cur NPs regulated neural stem cells differentiation in physiological condition. Red arrowed line: inflammatory cytokines induced GFAP^+^ astrocyte from stem cell was increased. Green arrowed line: RA@BSA@Cur NPs inhibited the astrocyte differentiation, enhanced the neuronal differentiation. (**b**) Immunofluorescence-stained NE4C differentiation for the control, RA@BSA, Cur@BSA, and RA@BSA@Cur groups in pathological condition. Neurons (β3-tubulin: green, yellow arrow) and astrocyte (GFAP: red), nuclei (DAPI: blue), (n = 3 independent samples). Scale bars, 50 μm. (**c**) The ratio of β3-tubulin^+^/GFAP^+^ NE4C treated with RA@BSA@Cur NPs via flow cytometry, and (**d**) quantitative analysis of β3-tubulin^+^/GFAP^+^ cells ratio for the groups. (**e**&**f**) The β3-tubulin and GFAP protein amounts change of NE4C via western blotting. (**g**) Immunofluorescence-stained primary NSC for the control, RA@BSA, Cur@BSA, and RA@BSA@Cur groups in pathological condition. Neurons (β3-tubulin: green, yellow arrow) and astrocytes (GFAP: red), nuclei (DAPI: blue). Scale bars, 50 μm. (**h**) Quantitative analysis of β3-tubulin/GFAP fluorescence intensity for all groups. (**i**) The quantitative analysis of β3-tubulin^+^ cell length. (n = 3 independent samples). Statistical differences were determined by using the ANOVA with Bonferroni's multiple comparison test (*p < 0.05, **p < 0.01, ***p < 0.001, ns: no significant; a.u. means arbitrary unit).

Directing the NSCs differentiation is critical to establish a permissive microenvironment for spinal cord regeneration [26,27]. Astrocytes can exhibit axon demyelination when insufficient neurons are present, whereas excessive astrocytes would form glial scar [28]. As inflammatory cytokines affect the differentiation of NSCs [29], the effect of RA@BSA@Cur NPs on the differentiation of NSCs at pathological conditions was further explored (Fig. 4a). The neuronal/astroglial differentiation behavior of the mice NSC line NE4C was investigated when it was cultured with RA@BSA@Cur NPs. NE4C cells exhibited high GFAP expression with LPS stimulation, suggesting glial scar formation under inflammatory conditions. Neural differentiation was not stimulated but astrocyte differentiation was suppressed the in the Cur@BSA group. RA@BSA@Cur NPs exhibited high β3-tubulin and low GFAP fluorescence intensity, suggesting that RA and Cur promoted the differentiation of NSCs synergistically under pathological conditions (Fig. 4b). The GFAP^+^ astrocyte ratio (approximately 35%) in the PBS + LPS group was higher than that in the Cur@BSA (approximately 28%) and RA@BSA@Cur (approximately 20%) groups (Fig. 4c–d). Moreover, these results were confirmed using western blotting. The levels of β3-tubulin were increased in the RA@BSA@Cur group compared with the PBS + LPS and Cur@BSA groups (Fig. 4e–f). Further, we extracted and identified primary mice NSCs (Fig. S10, Supporting Information). The LPS induction experiment was repeated on these primary mice NSCs. They tended to differentiate into GFAP^+^ astrocytes with LPS stimulation. More number of β3-tubulin^+^ neurons and lower number of GFAP^+^ astrocytes were observed in the RA@BSA@Cur group (Fig. 4g–h). In addition, the length of β3-tubulin^+^ cells in the RA@BSA@Cur group (72 ± 16.8 μm) was longer than that in the PBS + LPS group (10 ± 2.5 μm, p < 0.05) (Fig. 4i). Collectively, these results suggested that RA@BSA@Cur NPs promoted neuronal differentiation of NSCs under pathological conditions.

### 2.7 RA@BSA@Cur NPs reduced NSC-derived astrocytes through regulating M1/M2 macrophage polarization

At the acute stage of SCI, the levels of endogenous NSCs and macrophages are increased almost simultaneously at 3–7 days [30,31]. M1 macrophages influence stem cell differentiation, and the asymmetric polarization of macrophages induce endogenous NSC differentiation into mainly astrocytes in SCI [32]. RA@BSA@Cur NPs inhibited polarization of macrophages into M1 phenotype and reduced the secretion of proinflammatory factors. It should be further explored whether RA@BSA@Cur NPs reduced NSC-derived GFAP^+^ astrocytes through M1/M2 macrophage polarization (Fig. S11a, Supporting Information). RAW264.7 cells were cultured with various BSA-based NPs under LPS treatment. The cell supernatant was collected after centrifugation to induce NE4C differentiation, which was used to observe the astrocytic differentiation (Figs. S11b–11c, Supporting Information). The fluorescence intensity of GFAP in the RA@BSA@Cur group was reduced (7.57 ± 2.04), which was much lower than that in the PBS + LPS (22.01 ± 7.94) and RA@BSA (16.7 ± 6.51) groups. The fluorescence intensity of GFAP^+^ astrocytes in the RA@BSA@Cur group was comparable to that in the control group (6.84 ± 1.64). In addition, a similar phenomenon was observed in primary NSCs. Because of the fewer RA@BSA@Cur NPs in the RAW264.7 supernatant, the β3-tubulin^+^ neuron cells were few. Moreover, the levels of GFAP^+^ cells were reduced because of the decreasing inflammatory cytokines in RAW264.7 supernatant (Fig. S12, Supporting Information). Collectively, these results demonstrated that RA@BSA@Cur NPs played a key role in the glial scar formation via regulating polarization of macrophages around NSCs.

### 2.8 RA@BSA@Cur NPs enriched at spinal cord injury site

**Fig. 5:**
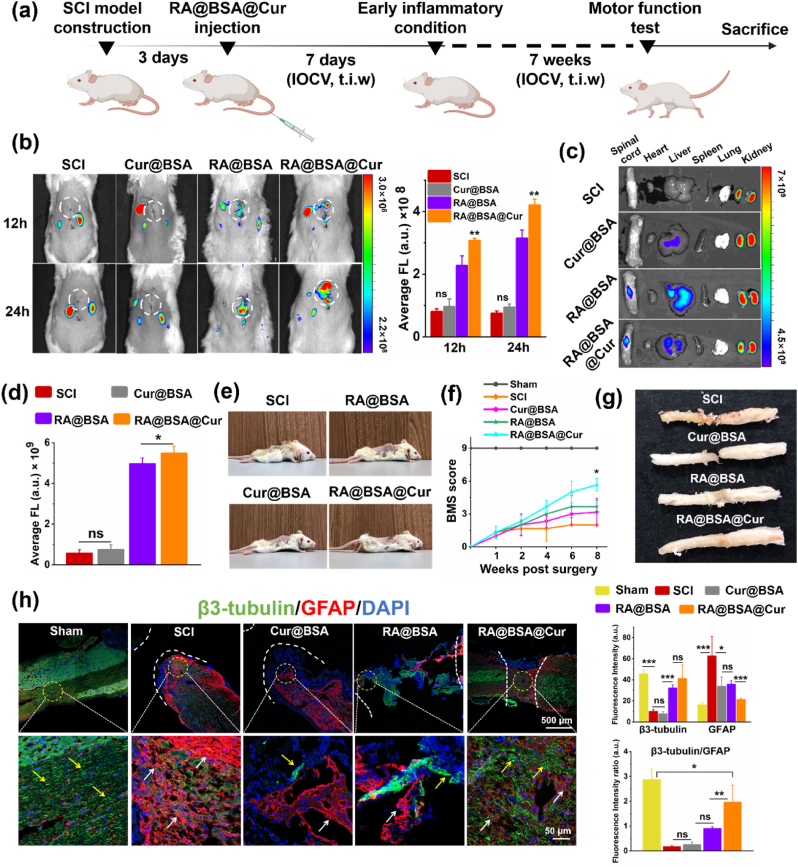
**Functional recovery of mice in different groups *in vivo*.** (**a**) Schedule of spinal cord injury mice treated with RA@BSA@Cur NPs. IOCV: Injection of caudal vein, t.i.w: three times a week. (**b**) Fluorescence imaging of SCI mice post i.v. Injection of Cy5.5-labeled RA@BSA, Cur@BSA, RA@BSA@Cur NPs *in vivo*. (**c**&**d**) Fluorescence imaging and qualitative analysis of spinal cord, heart, liver, spleen, lung and kidney in SCI mice post i.v. Injection after 48 h. (**e**) Behavior recovery of regenerated spinal cord injury mice. (**f**) Lower limb motor function over time using BMS score. (**g**) Macrography of spinal cord tissues at 8 weeks post-surgery. (**h**) Microscopic observation and quantitative assessment of the β3-Tubulin^+^ neurons (yellow arrow) and GFAP^+^ astrocytes (red arrow) in the defects after 8 weeks post-implantation. (n = 3 independent samples). Statistical differences were determined by using the ANOVA with Bonferroni's multiple comparison test (*p < 0.05, **p < 0.01, ***p < 0.001, ns: no significant; a.u. means arbitrary unit).

Administration of intravenous injection could reduce the risk of multiple intrathecal injections to the surrounding normal tissue [33]. Fluorescence imaging was used to assess the enrichment efficiency of RA@BSA@Cur NPs at the spinal cord after intravenous injection at the tail (Fig. 5a). To verify the biodistribution of RA@BSA@Cur NPs after labelling with Cy5.5, the *in vivo* fluorescence was measured at 12 and 24 h after intravenous injection (Fig. 5b). The red fluorescence signal at the SCI site was enhanced after RA@BSA@Cur NPs injection compared with that after Cur@BSA and RA@BSA NPs injections, probably because of renal clearance of the ultrasmall Cur@BSA and RA@BSA NPs (size <6 nm). SCI ruptures local blood vessels and induces inflammation at the trauma site, resulting in the increase in the permeability of blood vessels [34]. As the particle size of RA@BSA@Cur NPs was approximately 90 nm, it could penetrate blood vessels at the trauma site via EPR-like effect [35,36] and eventually accumulate at the SCI area. Overall, 48 h after injecting NPs, the mice were sacrificed, and the main organs and spinal cord were removed for ex vivo imaging. A strong fluorescence signal was detected at the SCI site, indicating good accumulation of RA@BSA@Cur NPs at SCI sites (Fig. 5c–d). To ensure that Cy5.5-RA@BSA@Cur NPs accumulated at SCI sites, the spinal cord tissue was observed by freezing sections and stained with DAPI. The fluorescence results indicated that Cy5.5-RA@BSA@Cur NPs (red fluorescence) were present at the SCI area and were rarely present in the normal spinal cord (Fig. S13, Supporting Information). In the spinal cord sections, the macrophages and NSCs were stained with specific antibodies, namely, CD68 and Nestin. It was observed that Cy5.5-RA@BSA@Cur NPs accumulated around the macrophages and NSCs (Fig. S14, Supporting Information). Collectively, these results suggested that RA@BSA@Cur NPs were enriched at SCI sites to affect endogenous nerve cells.

### 2.9 RA@BSA@Cur NPs improved mouse motor function after SCI

To evaluate the *in vivo* treatment of RA@BSA@Cur NPs, they were intravenously injected into spinal cord transection model mice. The sham, SCI, RA@BSA, and Cur@BSA NPs were used as the controls. The mice in the SCI, RA@BSA, and Cur@BSA groups faced paralysis over 2 months, whereas motor function restoration was observed in the RA@BSA@Cur group. The hind limbs motor confirmed the restorative function in the RA@BSA@Cur group 2 months after surgery (Fig. 5e). The mice in the RA@BSA@Cur group exhibited frequent plantar stepping, and their paws rotated at initial contact and lifted off. Sometimes, the tail went up and down (Video S1, Supporting Information). Basso mouse scale (BMS) tests were used to further analyze the locomotor function during 2 months after the RA@BSA@Cur treatment. The BMS scores increased gradually in all groups and showed various degrees of recovery. At 2 months after surgery, the BMS score of the SCI group was 2. Because of the introduction of the single drug loading BSA NPs (RA@BSA or Cur@BSA), a better locomotion recovery was achieved, and the score reached to 4. Surprisingly, the RA@BSA@Cur group exhibited the best functional recovery with BMS score of 6 (Fig. 5f, Video S1, Supporting Information); Moreover, this group exhibited better fine function recovery with BMS subscores above 5 (Fig. S15, Supporting Information). All mice were sacrificed after 2 months to study the histological structures of the regenerated nerve tissues. Cross-sectional wounds remained at the lesion sites in the SCI and Cur@BSA groups, and visible cavities and scar appeared in the RA@BSA group. However, these defects were reduced in the RA@BSA@Cur group (Fig. 5g). The motor function and macro image results indicated efficient functional recovery and tissue regeneration *in vivo* after treatment with RA@BSA@Cur NPs.

Supplementary data related to this article can be found at https://doi.org/10.1016/j.bioactmat.2022.05.026

The following is the supplementary data related to this article:

Multimedia component 2 Multimedia component 2

### 2.10 RA@BSA@Cur NPs balanced the *in vivo* neurons and astrocytes ratio

Astrocytes provide nutrients and protect neurons in CNS. However, astrocytes are activated by inflammation and form glial scars in SCI, which is a chemical barrier inhibiting the neurons regeneration [37]. Endogenous NSCs remain inactive in physiological environment and differentiate into astrocytes/neurons after SCI. The NSCs at lesion area could promote spinal cord regeneration; however, this process is often hindered by the presence of glial scar [30]. Considering the good neural/astrocytic differentiation behavior of RA@BSA@Cur NPs *in vitro*, the double-staining using β3-tubulin and GFAP was performed to analyze the distribution of neurons and astrocytes at the lesion sites (Fig. 5h). In the SCI and Cur@BSA groups, GFAP^+^ cells were aggregated at the lesion edge, and almost no neurons were present in the transaction edge. Although the lesion in the RA@BSA group was appropriately healed, significant cavity area still remained. Unlike the SCI, Cur@BSA, and RA@BSA groups, SCI treated with RA@BSA@Cur NPs exhibited a significantly higher number of neurons at the SCI site. Uniform distribution of neurons was accompanied by moderate distribution of astrocytes along the spinal cord. The β3-tubulin/GFAP fluorescence intensity ratio reached approximately 1.9, which was close to the ratio of glial cells and neurons (approximately 2.5) in the sham group (Fig. 5h). Collectively, these results demonstrated that RA@BSA@Cur NPs optimized the proportion of neurons and astrocytes for promoting regeneration of the spinal cord.

### 2.11 RA@BSA@Cur NPs effect the *in vivo* synaptic regeneration and scar formation

**Fig. 6:**
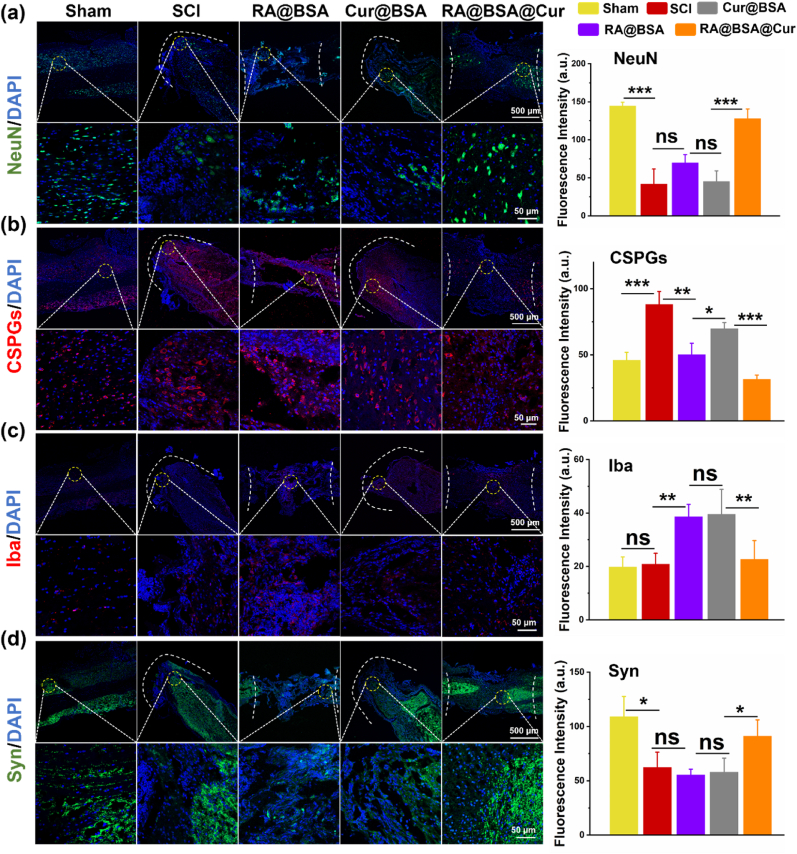
**RA@BSA@Cur NPs promoted synaptic regeneration and less scar formation *in vivo*.** Immunofluorescence staining and quantification analysis of NeuN ^+^ cells (**a**), CSPGs accumulation (**b**), Iba^+^ microglial cells (**c**), and synapsis remodeling (**d**). (n = 3 independent samples). Statistical differences were determined by using the ANOVA with Bonferroni's multiple comparison test (*p < 0.05, **p < 0.01, ***p < 0.001, ns: no significant; a.u. means arbitrary unit).

Spinal cord regeneration refers to several processes, such as neuronal maturation, scar hyperplasia, and synaptic reconstruction [38]. The local enrichment of RA@BSA@Cur and RA@BSA facilitated the neural differentiation of NSCs, where higher number of NeuN^+^ cells were distributed into the lesion sites than that in the control and Cur@BSA groups (Fig. 6a). The fluorescence intensity of chondroitin sulfate proteoglycans in the RA@BSA@Cur group was significantly lower than that in the other groups, indicating presence of less amount of scar tissue at the injury site (Fig. 6b). Interestingly, compared with the SCI and Cur@BSA groups, a small number of Iba^+^ microglial cells were present at the lesion sites in the RA@BSA@Cur group. This indicated that the reduced number of immune-associated cells might inhibit chronic inflammation in the middle and later periods of SCI (Fig. 6c) [39]. In addition, abundant synapse is critical for nerve regeneration and axon extension. As a critical synaptic signaling molecule, RA mediates the regulation of protein synthesis in the neuronal dendrites [40]. An increased synaptic distribution at lesion sites was observed after treatment with RA@BSA@Cur NPs (Fig. 6d). The expression of proteins associated with neuronal and glial scar was comparable in the RA@BSA@Cur and sham groups, demonstrating that RA@BSA@Cur NPs were favorable for the effective treatment of CNS-related diseases.

### 2.12 RA@BSA@Cur NPs modulated *in vivo* inflammation

**Fig. 7:**
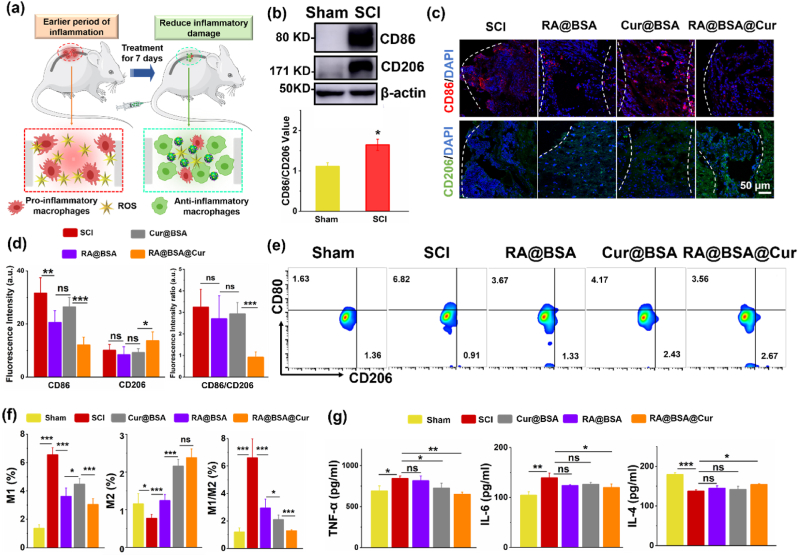
**RA@BSA@Cur NPs suppressed early inflammation after SCI**. (**a**) The schematic illustration of RA@BSA@Cur NPs inhibit early inflammation after SCI. (**b**) The protein change of CD86 and CD206 in normal and SCI. (**c**&**d**) Immunofluorescence staining and quantification analysis of CD86^+^ M1 and CD206^+^ M2 macrophage in sham, SCI, RA@BSA, Cur@BSA, and RA@BSA@Cur groups. Scale bars, 50 μm. (**e**&**f**) The ratio of CD80^+^/CD206^+^ cells in sham, SCI, RA@BSA, Cur@BSA, and RA@BSA@Cur groups. (**g**) The inflammatory-associated factors TNF-α, IL-6, and IL-4 changes in sham, SCI, RA@BSA, Cur@BSA, and RA@BSA@Cur groups. (n = 3 independent samples). Statistical differences were determined by using the ANOVA with Bonferroni's multiple comparison test (*p < 0.05, **p < 0.01, ***p < 0.001, ns: no significant; a.u. means arbitrary unit).

Furthermore, we assessed the effect of RA@BSA@Cur NPs on neural inflammation at the early stage of trauma (Fig. 7a). Western blotting results revealed that the expression of CD86, which is a common marker in M1 macrophages, was remarkably increased after SCI (Fig. 7b). Immunofluorescence imaging results suggested that RA@BSA@Cur NPs reduced the signal of CD86 and increased the signal of CD206 at the epicenter of SCI (Fig. 7c–d). The ratio of CD80^+^/CD206^+^ and the number of proinflammatory cells significantly reduced in the RA@BSA@Cur group (Fig. 7e–f). After RA@BSA@Cur treatment, the level of TNF-α and IL-6 significantly decreased and that of IL-4 increased (Fig. 7g). Thus, the inhibition of inflammation at the early stage of SCI could be decreased, and an environment for the induced regeneration of the spinal cord could be created [41]. H&E staining and histological analysis revealed no adverse effects and damages in tissues in all groups (Fig. S16, Supporting Information). H&E staining results indicated that RA@BSA@Cur NPs did not exhibit obvious long-term side effects in mice, suggesting their biomedical applications *in vivo*. Collectively, the results indicated that RA@BSA@Cur NPs established multifunctional microenvironments to induce the differentiation of NSCs and simultaneously inhibit neural inflammation, which supported the spinal cord regeneration with less scar and functional recovery.

## 3 Conclusion

In summary, RA@BSA@Cur NPs were synthesized via self-assembly method for the treatment of SCI. Cur, as an antioxidant drug, facilitated ROS scavenging, and RA enhanced survival of neurocytes and formation of neurite extensions. RA@BSA@Cur NPs could influence the proliferation and polarization ratio of M1/M2 macrophages and stem cells differentiation *in vitro*, demonstrating the synergistic treatment strategy for neuro-associated cells under pathological conditions. The *in vivo* experiments revealed that RA@BSA@Cur NPs could regulate the phenotypic polarization of macrophages, inhibit the release of inflammatory mediators, promote functional neuron regeneration, and reduce scar tissue formation. Therefore, this BSA-based drug delivery system provided a new nanoplatform that inhibited acute inflammation and tissue destruction and supported the recovery of functional neurons by forming a pro-regenerative microenvironment. This study unraveled novel avenues for biomimetic nanomaterials to treat CNS-related disorders, including SCI.

## 4 Experimental section

*Synthesis and Characterization of RA@BSA@Cur NPs:* Bovine Serum Albumin (BSA, B2064-10G, SIGMA) was labeled with retinoic acid (RA, Q107856-5g, D&B Biological Science and Technology Co.Ltd) and curcumin (Cur, S19245-5g, Shanghai Yuanye Bio-Technology Co., Ltd) through self-assembly method. In brief, ethanolic solution of 100 μL RA (3 mg/mL) and 100 μL Cur (3 mg/mL) were added into the solution of BSA (60 mg/mL, 1 mL, pH 8.0, adjusted with 0.1 M NaOH). Glutaraldehyde solution (20 μL) was added into the mixture to induce crosslinking. The mixture was then reacted for 24 h at room temperature. Excess unbound drugs were removed by centrifugation filtration through Amicon filters (MWCO = 10 kDa) and washed three times with PBS until no drugs in the filtration solution. Using the transmission electron microscope (FEI Tecnai F20), the morphology of RA@BSA@Cur NPs was characterized. The diameters of BSA and BSA-drugs were detected by Zeta sizer Nano-ZS (Malvern Instruments, UK). The combination and loading efficiency of drugs with BSA were detected by ultraviolet spectrophotometer.

*Quantitative Analysis of the Elimination of •OH and*^*1*^*O*_*2*_*:* The elimination of ^1^O_2_ was quantified by TEMPO an electron spin resonance (ESR) spectrometer (Bruker EMX plus). 1 mL of RA@BSA@Cur NPs solution with 100 μM H_2_O_2_ was mixed with 10 μL DMPO (1 M) for 30 s. The ESR spectrometer was used to test the characteristic peak signals. The ESR spectrometer settings: center field, 3520 G; sweep width, 100 G; microwave frequency, 9.77 GHz; modulation frequency, 100 kHz; power, 20.00 mW. The change of •OH with H_2_O_2_ catalytic decomposition by RA@BSA@Cur NPs was detected by the TMB reagents color-developing method. 4 μL TMB (80 mM) was added into 1 mL of RA@BSA@Cur solution with 100 μM H_2_O_2_. The absorbance changes of TMB at 654 nm were monitored to assess the elimination of •OH. The ABTS^•+^ change was determined by ABTS reagents color-developing method. The antioxidant capacity of RA@BSA@Cur NPs was evaluated by the absorbance change at 414 nm by the UV–vis spectrophotometer.

*RAW264.7 cells Behaviors by RA@BSA@Cur NPs:* The BSA-related nanomaterials were flited by 0.2 μm filtration to remove bacteria, and then used to co-culture with macrophage cells. Mice macrophage line RAW264.7 cells were purchased from Wuhan Procell Life Science & Technology Co.,Ltd. RAW264.7 cells (2.0 × 10^4^ cells per well) were seed on 24-well plate with DMEM medium containing 10% FBS, and treated with different BSA nanoparticles or LPS (100 ng/mL) in specified time. After stimulation, the samples were then stained with CD86 (Abcam, ab239075, USA) or CD206 (Anti-Mannose Receptor antibody, Abcam, ab125028, USA), and DAPI (Invitrogen, 62247, USA). The polarization ratio of the macrophage was observed by Confocal Laser Scanning Microscopy (CLSM, LSM 800 with Airyscan, ZEISS, Germany). In a similar way, multiple BSA nanoparticles or LPS (100 ng/mL) were used to induce the RAW264.7 cells in 12-well plate. After 24 h, the single-cell suspension was prepared in equilibrium liquid, stained with antibodies, FITC-F4/80, APC-CD80, PE-CD206 at 4 °C for 1 h. Flow cytometry (C6 Plus Flow Cytometry, BD Accuri, USA) was used for analyzing ratio of M1/M2 cells polarization.

As for Western blot analysis, macrophage was cultured in 6-well plate, the treatment method followed the preceding step, then the sodium dodecyl sulfate polyacrylamide gel electrophoresis was used to separate protein, and a polyvinylidene fluoride membrane transferred proteins. The membranes were incubated with CD86 (Abcam, ab239075, San Francisco, USA), I*κ*B*α* (Affinity, AF6239, USA), phospho-I*κ*B*α* (Affinity, AF2002, USA), NF-*κ*B p65 (Affinity, AF5006, USA), phosphor-NF-*κ*B p65 (Affinity, AF2006, USA), and corresponding secondary antibody (Goat Anti-Rabbit IgG H + L HRP, Affinity, S0001, USA) respectively. The targeted protein bands were visualized on X-ray film via the enhanced chemiluminescence reagents (Bi Yuntian, Wuxi, China). The band gray value was quantified by the ImageJ software. The secretion of TNF-α (mlbio, ml002095, Shanghai, China), IL-6 (mlbio, ml002293, Shanghai, China) and IL-4 (mlbio, ml063156-J, Shanghai, China) from macrophage were quantified by ELISA kits.

*Reactive oxygen species scavenging of RA@BSA@Cur in BV2 Cell:* For microglial line BV2 cells (Shanghai Institutes for Biological Science), the cells were incubated with different BSA nanoparticles for or LPS (100 ng/mL) in 24 h. After stimulation, the cells were stained with 2′,7′-dichlorofluorescent yellow diacetate (DCFH-DA, 20 μM) for 30 min, to qualitative analysis ROS scavenging efficiency.

*Cytocompatibility and Endocytosis of RA@BSA@Cur to Schwann Cells and PC12 cells:* Schwann cells (Shanghai Institutes for Biological Science) and PC12 cells (Wuhan Procell Life Science & Technology Co.,Ltd) were seeded in 96-well plates with 2000 cells density per well, and the different concentrations of RA@BSA, Cur@BSA, and RA@BSA@Cur NPs were added to wells for 48 h. Cell counting kit-8 (CCK-8) assay was used to determine the Schwann and PC12 cells proliferation activity. 2 μL of NHS-Cy5.5 (10 mg/mL) was added to RA@BSA@Cur solution and stirred for 24 h. Amicon filters (MWCO = 10 kDa) was used to remove the free Cy5.5 via centrifugation filtration, until no detachable color in filtration solution. Then the Cy5.5 labeled-RA@BSA@Cur was co-incubated with Schwann cells and PC12 cells for 6 h, the material positioning in cells was observed by Confocal Laser Scanning Microscopy (CLSM, LSM 800 with Airyscan, ZEISS, Germany).

*Proliferation and Axon Spreading of PC12 Cells with RA@BSA@Cur nanoparticle:* Low differentiation PC12 cells were prepared. 2.0 × 10^4^ PC12 cells mL^−1^ were cultured in 24-well plate with DMEM medium containing 10% FBS, treated with different concentration RA@BSA@Cur nanoparticles, and then stained with F-actin (FITC-phalloidin, Invitrogen, B1370, USA) and DAPI to choose the suitable concentration for further experiments. The PC12 cells treat with RA@BSA, Cur@BSA and RA@BSA@Cur NPs, then stained with FITC-phalloidin (Invitrogen, B1370, USA), and DAPI. The PC12 cells were incubated with different BSA nanoparticles or LPS (100 ng/mL) in 24 h, The cells were stained with FITC-phalloidin to detect the neurite length change in pathological condition. The neurite length change of the PC12 cells was visualized by CLSM.

*The extraction and identification of primary Neural Stem Cell:* According to reported method [42], the mice embryos were used to extract primary NSCs. The NSC culture medium formula: 90% DMEM/F12 (Gibco, A4192001, USA) with 10% B27 (Invitrogen, 17504044, USA), 20 ng/mL epidermal growth factor (Gibco, PHG0311, USA), 20 ng/mL basic fibroblast growth factor (PeproTech, 400–29, USA). The NSC spheres were identified with anti-nestin antibody (Affinity Biosciences, DF7754, USA), anti-β3-tubulin antibody (Abcam, ab78078, USA), and anti-MAP2 antibody (Affinity Biosciences, AF4081, USA) respectively, then labeled with secondary antibody (Alexa 488, Abcam, ab150077, USA) and DAPI.

*Neural Stem Cell Differentiation with RA@BSA@Cur NPs:* Neural stem cells (NSCs) line NE4C cells was purchased from Wuhan Procell Life Science & Technology Co.,Ltd. The NE4C cells and primary NSCs were incubated in DMEM/F12 containing 10% FBS, treated with different BSA nanoparticles or LPS (100 ng/mL). After stimulation, the samples were stained with anti-GFAP primary antibody (Abcam, ab7260, USA) and anti-β3-tubulin primary antibody (Abcam, ab78078, USA), then stained with Alexa 488 or Alexa647 secondary antibody, finally stained with DAPI. The neurons and astrocytes differentiation were observed with CLSM. The single-cell suspension was prepared, and stained with GFAP and β3-tubulin antibodies. Flow cytometry was used for analyzing ratio of neurons/astrocytes differentiation proportion. Followed the preceding treatment method, the membranes were incubated with GFAP, β3-tubulin primary antibodies, and corresponding secondary antibody respectively. Western blot was used to analyze protein expression. The effect of RA@BSA@Cur NPs on relationship between macrophage-derived inflammatory factor with neural stem cells was explored. The RAW264.7 cultured with different BSA-based NPs in LPS condition. The cell supernatant was centrifuged and collected to induce NE4C and primary NSCs. The neurons/astrocytes proportion ratio of the NE4C and primary NSCs was observed by CLSM.

*Animal Experiments of Spinal Cord Injury:* The mice spinal cord transection model was used to evaluate tissue regeneration [41]. Ethics Committee (Second Affiliated Hospital of Soochow University) has approved the animal experiments. Six-week-old female BALB/c mice (20 ± 1 g, n = 45, provided by Soochow University Experimental Animal Center) were divided into five groups. The mice treated with RA@BSA, Cur@BSA, and RA@BSA@Cur NPs by tail vein injection were termed as RA@BSA, Cur@BSA, and RA@BSA@Cur groups, respectively. The mice with transaction defects with normal saline injection were SCI group, and the mice without spinal cord injury were the sham group. The nanoparticle solution was sterilized by filtration before animal experiments. 1% pentobarbital sodium (125 μL/20 g) was used to anesthetize mice via intraperitoneal injection. The 1.5 cm incision of skin center was lanced on T9-T10. The spinous process and vertebral plate were separated and exposed. The surgical knife blade was used to across T9 spinal cord for complete transverse injury model. The muscle and skin were sutured layer-by-layer. Each mouse was intramuscular injected with antibiotics for 3 days. It is reported that endogenous neural stem cells and macrophages were increased and at peak 3–7 days after spinal cord injury [30,31,43]. The M1 macrophages secreted pro-inflammatory factor, induced stem cells differentiate into astrocytes. On the contrary, the M2 macrophages could induce stem cells to differentiate into neuron. In consequence, either RA@BSA, Cur@BSA, RA@BSA@Cur nanoparticles (150 μL, 2 mg/mL) or normal saline (150 μL) were injected through tail vein injection 3 days after SCI, to affect more endogenous neural stem cells and macrophages. The nanoparticles and normal saline were injected three times a week (i.v. injection), which lasted until week 8.

*RA@BSA@Cur NPs Targeted to Spinal Cord Injury in Imaging:* For fluorescence imaging, the different BSA nanoparticles-Cy5.5 NSs (150 μL, 2 mg/mL) or free Cy5.5 solution was intravenously (i.v.) injected. The mice were imaged *in vivo* at different time points (12, 24 h) by Maestro EX fluorescence imaging system. The mice were deep euthanized after 48 h, and the spinal cord, heart, liver, spleen, lung and kidney were collected for *in vitro* imaging.

*Animal Motor Function after RA@BSA@Cur injection:* Hindlimb recovery was assessed with Basso Mouse Scale (BMS) score and subscore [44]. The BMS score (ranges from 0 to 9) was used to detect hind limb locomotive function change. BMS subscore (ranges from 0 to 11) was as follow: plantar stepping; paw position; coordination; tail position and trunk stability [45]. The BMS scores of the mice were observed and recorded weekly. In the eighth week, the movement trace of the treated group was also videoed. The hind limb movement and weight-bearing ability were recorded when mice walked on horizontal plane. The observers were blinded to the SCI mice condition, and the score evaluations were repeated five times and recorded. All recorders were trained in advance. The mice were evaluated at week 0, 1, 2, 4, 6, 8.

*Immunofluorescence analyses:* In the eighth week post-operation, the mice were deep euthanized. The mice were intracardially perfused with saline solution (50 mL) and 4% paraformaldehyde (50 mL). Then the spinal cord containing the lesion area was dissected and dewatered in 30% sucrose solution. The spinal cord in each group was photographed with digital camera, followed by embedded in OCT-freeze medium. The samples were cut into 8 μm thick sections along the tissue sagittal axis. Immunofluorescent staining was used to identify β3-tubulin (Abcam, ab78078, USA), GFAP (Abcam, ab7260, USA), NeuN (Abcam, ab177487, USA), CSPG (Affinity, DF7561, USA), Iba (Abcam, ab178846, USA) and Synaptophysin (Abcam, ab32127, USA) at the lesion area.

*Inflammation analyses in vivo:* Western blot was used to analyze CD86/CD206 expression in sham and SCI groups. One week after BSA NPs treatment, the spinal cord tissue was dissected and embedded in OCT-freeze medium. The sections in each group were stained with CD86 and CD206 at the lesion area. In addition, the single-cell suspension was prepared from injured tissue, stained with FITC-F4/80, APC-CD80, PE-CD206, followed by flow cytometry detection. The TNF-α, IL-6 and IL-4 in SCI site were quantified by ELISA kits.

*Statistical Methods:* All experimental data are represented as mean ± standard deviation (SD). One-way analysis of variance (ANOVA) followed by Tukey's multiple comparison test was used to evaluate the results. The difference between the groups was compared by GraphPad Prism Software. The p < 0.05 was considered statistically significant.

## Declaration of interests

The authors declare that they have no known competing financial interests or personal relationships that could have appeared to influence the work reported in this paper.

## References

[1] Holmes D. (2017). Spinal-cord injury: spurring regrowth. Nature.

[2] Huang H., Young W., Skaper S., Chen L., Moviglia G., Saberi H., Al-Zoubi Z., Sharma H.S., Muresanu D., Sharma A., El Masry W., Feng S. (2020). N. International association of, N. The Chinese association of, clinical neurorestorative therapeutic guidelines for spinal cord injury (IANR/CANR version 2019). J Orthop Translat.

[3] Milich L.M., Ryan C.B., Lee J.K. (2019). The origin, fate, and contribution of macrophages to spinal cord injury pathology. Acta Neuropathol..

[4] Marshall J., Zhou X.Z., Chen G., Yang S.Q., Li Y., Wang Y., Zhang Z.Q., Jiang Q., Birnbaumer L., Cao C. (2018). Antidepression action of BDNF requires and is mimicked by Galphai1/3 expression in the hippocampus. Proc. Natl. Acad. Sci. U. S. A..

[5] Tan K., Koyama S., Sakurai H., Teranishi T., Kanada Y., Tanabe S. (2021). Wearable robotic exoskeleton for gait reconstruction in patients with spinal cord injury: a literature review. J Orthop Translat.

[6] Gaudet A.D., Fonken L.K. (2018). Glial cells shape pathology and repair after spinal cord injury. Neurotherapeutics.

[7] Huang Y., Ren H., Gao X., Cai D., Shan H., Bai J., Sheng L., Jin Y., Zhou X. (2021).

[8] Kobayakawa K., Ohkawa Y., Yoshizaki S., Tamaru T., Saito T., Kijima K., Yokota K., Hara M., Kubota K., Matsumoto Y., Harimaya K., Ozato K., Masuda T., Tsuda M., Tamura T., Inoue K., Edgerton V.R., Iwamoto Y., Nakashima Y., Okada S. (2019). Macrophage centripetal migration drives spontaneous healing process after spinal cord injury. Sci. Adv..

[9] Zhou X., Wahane S., Friedl M.S., Kluge M., Friedel C.C., Avrampou K., Zachariou V., Guo L., Zhang B., He X., Friedel R.H., Zou H. (2020). Microglia and macrophages promote corralling, wound compaction and recovery after spinal cord injury via Plexin-B2. Nat. Neurosci..

[10] Deng W., Shao F., He Q., Wang Q., Shi W., Yu Q., Cao X., Feng C., Bi S., Chen J., Ma P., Li Y., Gong A., Tong S., Yu J., Spector M., Xu X., Zhang Z. (2019). EMSCs build an all-in-one niche via cell-cell lipid raft assembly for promoted neuronal but suppressed astroglial differentiation of neural stem cells. Adv. Mater..

[11] Beck K.D., Nguyen H.X., Galvan M.D., Salazar D.L., Woodruff T.M., Anderson A.J. (2010). Quantitative analysis of cellular inflammation after traumatic spinal cord injury: evidence for a multiphasic inflammatory response in the acute to chronic environment. Brain.

[12] Li X., Wei Z., Wang X., Duan F., Xiong L., Li J., Tian J., Jia L., Gao H. (2021). Premna microphylla Turcz leaf pectin exhibited antioxidant and anti-inflammatory activities in LPS-stimulated RAW 264.7 macrophages. Food Chem..

[13] Yuan Y., Chen Y., Peng T., Li L., Zhu W., Liu F., Liu S., An X., Luo R., Cheng J., Liu J., Lu Y. (2019). Mitochondrial ROS-induced lysosomal dysfunction impairs autophagic flux and contributes to M1 macrophage polarization in a diabetic condition. Clin. Sci. (Lond.).

[14] Gao Z.S., Zhang C.J., Xia N., Tian H., Li D.Y., Lin J.Q., Mei X.F., Wu C. (2021). Berberine-loaded M2 macrophage-derived exosomes for spinal cord injury therapy. Acta Biomater..

[15] Bai J., Zhang Y., Zheng X., Huang M., Cheng W., Shan H., Gao X., Zhang M., Sheng L., Dai J., Deng Y., Zhang H., Zhou X. (2020). LncRNA MM2P-induced, exosome-mediated transfer of Sox9 from monocyte-derived cells modulates primary chondrocytes. Cell Death Dis..

[16] Jung K.K., Lee H.S., Cho J.Y., Shin W.C., Rhee M.H., Kim T.G., Kang J.H., Kim S.H., Hong S., Kang S.Y. (2006). Inhibitory effect of curcumin on nitric oxide production from lipopolysaccharide-activated primary microglia. Life Sci..

[17] Wang F., Xia J.J., Shen L.J., Jiang T.T., Li W.L., You D.L., Chang Q., Hu S.Y., Wang L., Wu X. (2022). Curcumin attenuates intracerebral hemorrhage-induced neuronal apoptosis and neuroinflammation by suppressing the JAK1/STAT1 pathway. Biochem. Cell. Biol..

[18] Chen Y., Yuan F., Lin J., Zhang X., Luo J., Huang L. (2021). Curcumin promotes the proliferation, invasion of neural stem cells and formation of neurospheres via activating SDF-1/CXCR4 axis. Folia Neuropathol..

[19] Maden M. (2007). Retinoic acid in the development, regeneration and maintenance of the nervous system. Nat. Rev. Neurosci..

[20] Spada A., Emami J., Tuszynski J.A., Lavasanifar A. (2021). The uniqueness of albumin as a carrier in nanodrug delivery. Mol. Pharm..

[21] Jiang Y., Stenzel M. (2016). Drug delivery vehicles based on albumin-polymer conjugates. Macromol. Biosci..

[22] Belatik A., Hotchandani S., Bariyanga J., Tajmir-Riahi H.A. (2012). Binding sites of retinol and retinoic acid with serum albumins. Eur. J. Med. Chem..

[23] Tello Velasquez J., Nazareth L., Quinn R.J., Ekberg J.A., St John J.A. (2016). Stimulating the proliferation, migration and lamellipodia of Schwann cells using low-dose curcumin. Neuroscience.

[24] Roth J.A., Rosenblatt T., Lis A., Bucelli R. (2001). Melatonin-induced suppression of PC12 cell growth is mediated by its Gi coupled transmembrane receptors. Brain Res..

[25] Li Y., Liu L., Yu Z., Yu Y., Sun B., Xiao C., Luo S., Li L. (2021). Effects of edaravone on functional recovery of a rat model with spinal cord injury through induced differentiation of bone marrow mesenchymal stem cells into neuron-like cells. Cell. Reprogr..

[26] Fan B., Wei Z., Yao X., Shi G., Cheng X., Zhou X., Zhou H., Ning G., Kong X., Feng S. (2018). Microenvironment imbalance of spinal cord injury. Cell Transplant..

[27] Wang S., Jeffries E., Gao J., Sun L., You Z., Wang Y. (2016). Polyester with pendent acetylcholine-mimicking functionalities promotes neurite growth. ACS Appl. Mater. Interfaces.

[28] Domingues A.V., Pereira I.M., Vilaca-Faria H., Salgado A.J., Rodrigues A.J., Teixeira F.G. (2020). Glial cells in Parkinson s disease: protective or deleterious?. Cell. Mol. Life Sci..

[29] Kameda T., Imamura T., Nakashima K. (2018). Epigenetic regulation of neural stem cell differentiation towards spinal cord regeneration. Cell Tissue Res..

[30] Stenudd M., Sabelstrom H., Frisen J. (2015). Role of endogenous neural stem cells in spinal cord injury and repair. JAMA Neurol..

[31] Gensel J.C., Zhang B. (2015). Macrophage activation and its role in repair and pathology after spinal cord injury. Brain Res..

[32] Ma Y., Deng M., Liu M. (2019). Effect of differently polarized macrophages on proliferation and differentiation of ependymal cells from adult spinal cord. J. Neurotrauma.

[33] Haroutiunian S., Kagan L., Yifrach-Damari I., Davidson E., Ratz Y., Hoffman A. (2014). Enhanced antinociceptive efficacy of epidural compared with i.v. methadone in a rat model of thermal nociception. Br. J. Anaesth..

[34] Jin L.Y., Li J., Wang K.F., Xia W.W., Zhu Z.Q., Wang C.R., Li X.F., Liu H.Y. (2021). Blood-spinal cord barrier in spinal cord injury: a review. J. Neurotrauma.

[35] Tan X., Luo S., Long L., Wang Y., Wang D., Fang S., Ouyang Q., Su Y., Cheng T., Shi C. (2017). Structure-guided design and synthesis of a mitochondria-targeting near-infrared fluorophore with multimodal therapeutic activities. Adv. Mater..

[36] Xiao Q., Li X., Li Y., Wu Z., Xu C., Chen Z., He W. (2021). Biological drug and drug delivery-mediated immunotherapy. Acta Pharm. Sin. B.

[37] D'Ambrosi N., Apolloni S. (2020). Fibrotic scar in neurodegenerative diseases. Front. Immunol..

[38] Yang Y., Fan Y., Zhang H., Zhang Q., Zhao Y., Xiao Z., Liu W., Chen B., Gao L., Sun Z., Xue X., Shu M., Dai J. (2021). Small molecules combined with collagen hydrogel direct neurogenesis and migration of neural stem cells after spinal cord injury. Biomaterials.

[39] Li Y., Ritzel R.M., Khan N., Cao T., He J., Lei Z., Matyas J.J., Sabirzhanov B., Liu S., Li H., Stoica B.A., Loane D.J., Faden A.I., Wu J. (2020). Delayed microglial depletion after spinal cord injury reduces chronic inflammation and neurodegeneration in the brain and improves neurological recovery in male mice. Theranostics.

[40] Chen L., Lau A.G., Sarti F. (2014). Synaptic retinoic acid signaling and homeostatic synaptic plasticity. Neuropharmacology.

[41] Li Y., He X., Kawaguchi R., Zhang Y., Wang Q., Monavarfeshani A., Yang Z., Chen B., Shi Z., Meng H., Zhou S., Zhu J., Jacobi A., Swarup V., Popovich P.G., Geschwind D.H., He Z. (2020). Microglia-organized scar-free spinal cord repair in neonatal mice. Nature.

[42] Chen D., Hu S., Wu Z., Liu J., Li S. (2018). The role of MiR-132 in regulating neural stem cell proliferation, differentiation and neuronal maturation. Cell. Physiol. Biochem..

[43] McTigue D.M., Wei P., Stokes B.T. (2001). Proliferation of NG2-positive cells and altered oligodendrocyte numbers in the contused rat spinal cord. J. Neurosci..

[44] Wang C., Zhang L., Ndong J.C., Hettinghouse A., Sun G., Chen C., Zhang C., Liu R., Liu C.J. (2019). Progranulin deficiency exacerbates spinal cord injury by promoting neuroinflammation and cell apoptosis in mice. J. Neuroinflammation.

[45] Khayrullina G., Bermudez S., Byrnes K.R. (2015). Inhibition of NOX2 reduces locomotor impairment, inflammation, and oxidative stress after spinal cord injury. J. Neuroinflammation.

