# Supplementary material for: An anti-inflammatory and neuroprotective biomimetic nanoplatform for repairing spinal cord injury
Source: Bioact Mater. 2022 Jun 2;18:569–82. doi: 10.1016/j.bioactmat.2022.05.026 (PMC9256979; doi:10.1016/j.bioactmat.2022.05.026)
Supplement: Multimedia component 1 [file mmc1.docx]

**An Anti-inflammatory and Neuroprotective Biomimetic Nanoplatform for**

**Repairing Spinal Cord Injury**

Xiang Gao^1,2^, Zhihui Han^2^, Cheng Huang^1,2^, Huali Lei^2^, Guangqiang Li^2^, Lin Chen^1^, Dandan Feng^1^, Zijie Zhou^1^, Qin Shi^3^, Liang Cheng^2^* and Xiaozhong Zhou^1^*

*^1^* Department of Orthopedics, The Second Affiliated Hospital of Soochow University,

Suzhou, Jiangsu 215004, China

*^2^* Institute of Functional Nano & Soft Materials (FUNSOM), Collaborative Innovation Center of Suzhou Nano Science and Technology, Soochow University, Suzhou, Jiangsu, 215123, China

*^3^*Department of Orthopedics, the First Affiliated Hospital of Soochow University, Orthopedic Institute, Soochow University, 188 Shizi Road, 215006 Suzhou, Jiangsu, P. R. China

Corresponding authors: [zhouxz@suda.edu.cn](mailto:zhouxz@suda.edu.cn); [lcheng2@suda.edu.cn](mailto:lcheng2@suda.edu.cn)

**^†^** X. Gao, Z. Han, and C. Huang contributed equally to this work.

**Supporting Figures**


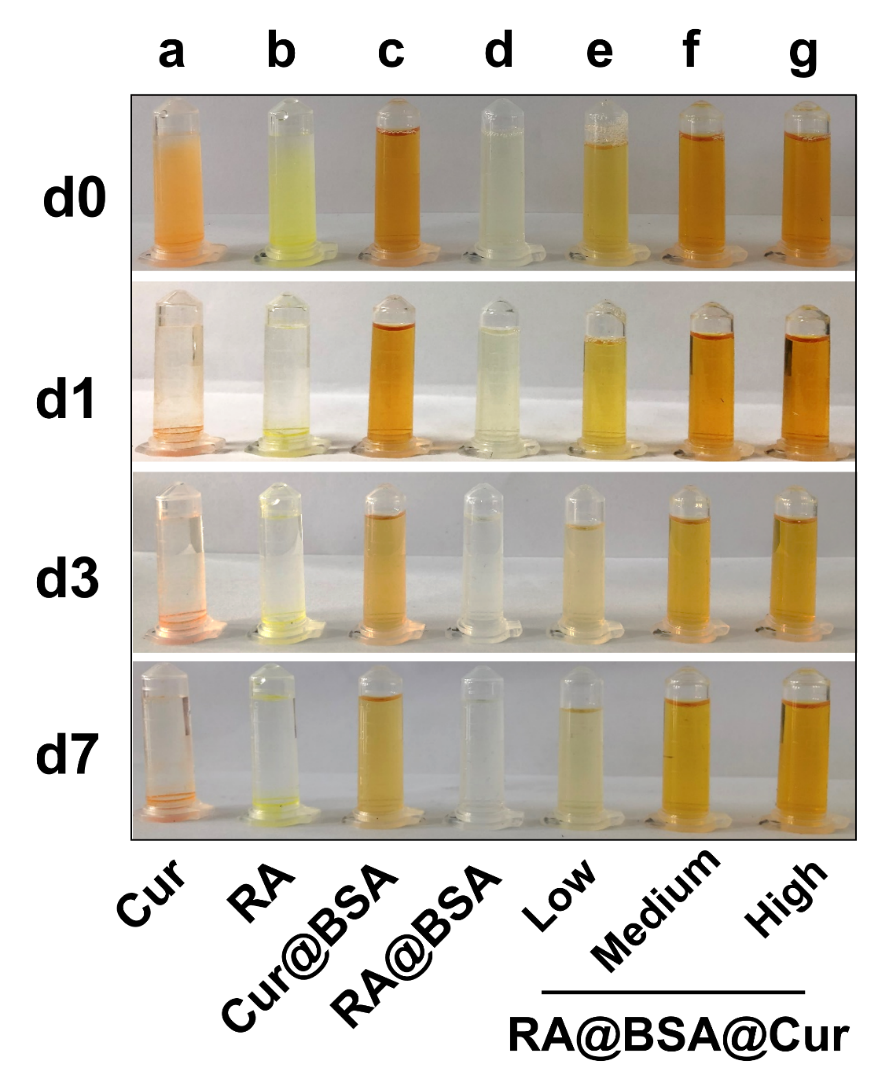


**Figure S1. The stability macrograph of drugs and drug-loaded BSA NPs in ultra-pure water at 0, 1, 3, 7 days.** The order of sample from left to right is: Cur; RA; Cur@BSA NPs; RA@BSA NPs; Low concentration of RA@BSA@Cur NPs (Low); Medium concentration of RA@BSA@Cur NPs (Medium); High concentration of RA@BSA@Cur NPs (High).


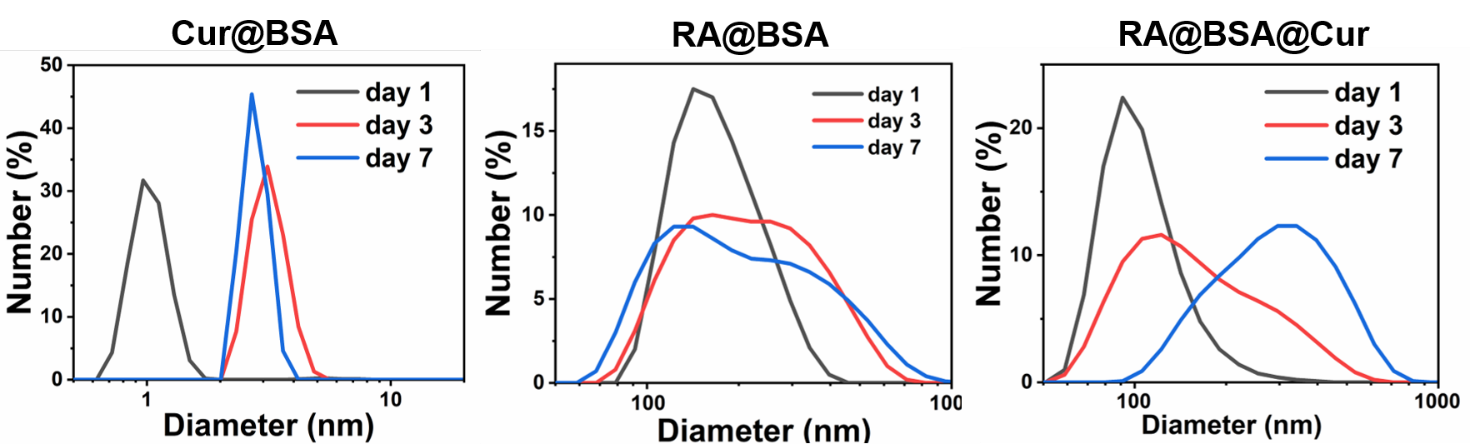


**Figure S2.** The size change of Cur@BSA, RA@BSA, RA@BSA@Cur NPs in ultra-pure water at 1, 3, and 7 days.


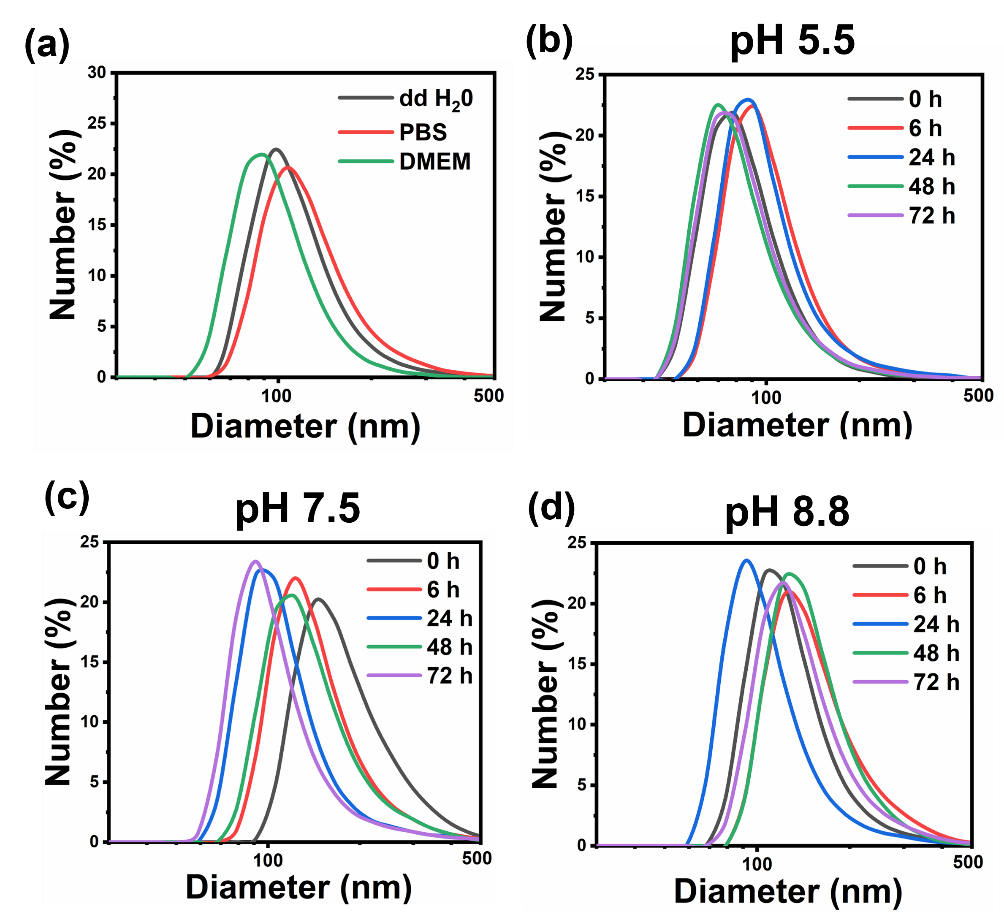


**Figure S3. The size change of RA@BSA@Cur NPs in different solutions.** (**a**) The stability of RA@BSA@Cur NPs in ultra-pure water, PBS, and DMEM at 3 days. (**b-d**) The size change of RA@BSA@Cur NPs in different pH PBS solutions at 0, 6, 24, 48, and 72 h.


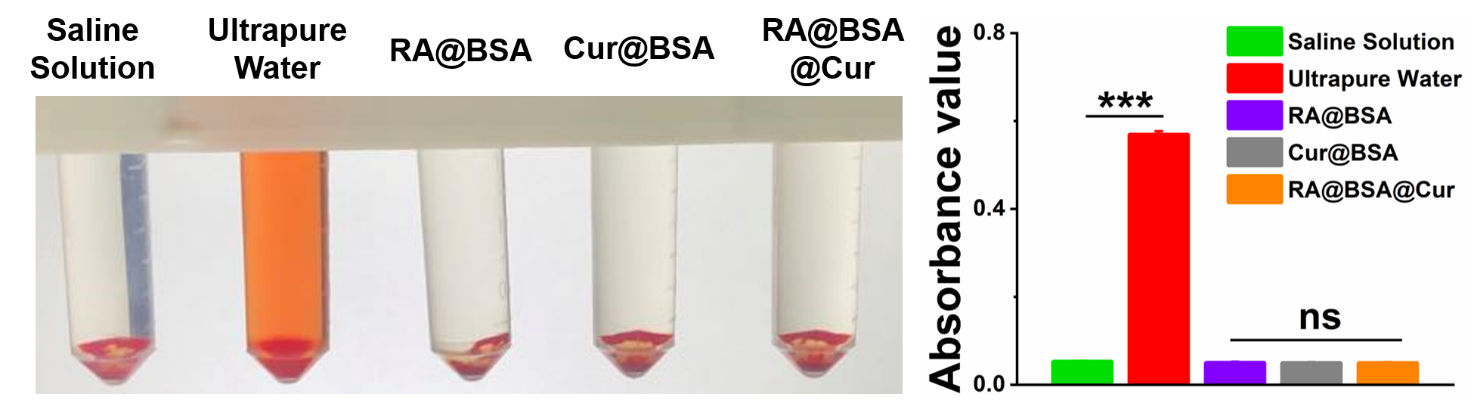


**Figure S4. The hemolytic test of RA@BSA@Cur NPs**. The macrograph of erythrocyte swelling changes in different groups (left graph). The absorbance quantification analysis of supernatant in groups (right graph). Statistical differences were determined by using the Analysis of Variance (ANOVA) with Bonferroni’s multiple comparison test (*p < 0.05, **p < 0.01, ***p < 0.001, ns: no significant; a.u. means arbitrary unit).


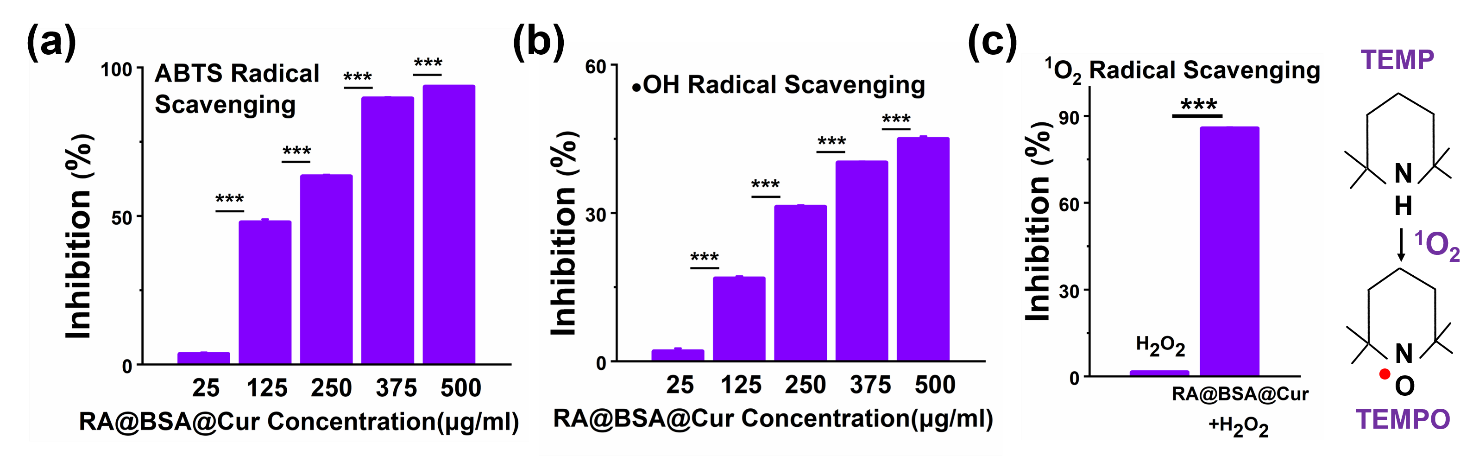


**Figure S5. ROS scavenging of RA@BSA@Cur NPs.** (**a**) ABTS^•+^ and (**b**) •OH scavenging ability of RA@BSA@Cur NPs in different concentrations. (**c**) ^1^O_2_ scavenging capacities of RA@BSA@Cur NPs. (n=3 independent samples). Statistical differences were determined by using the ANOVA with Bonferroni’s multiple comparison test (*p < 0.05, **p < 0.01, ***p < 0.001, ns: no significant; a.u. means arbitrary unit).


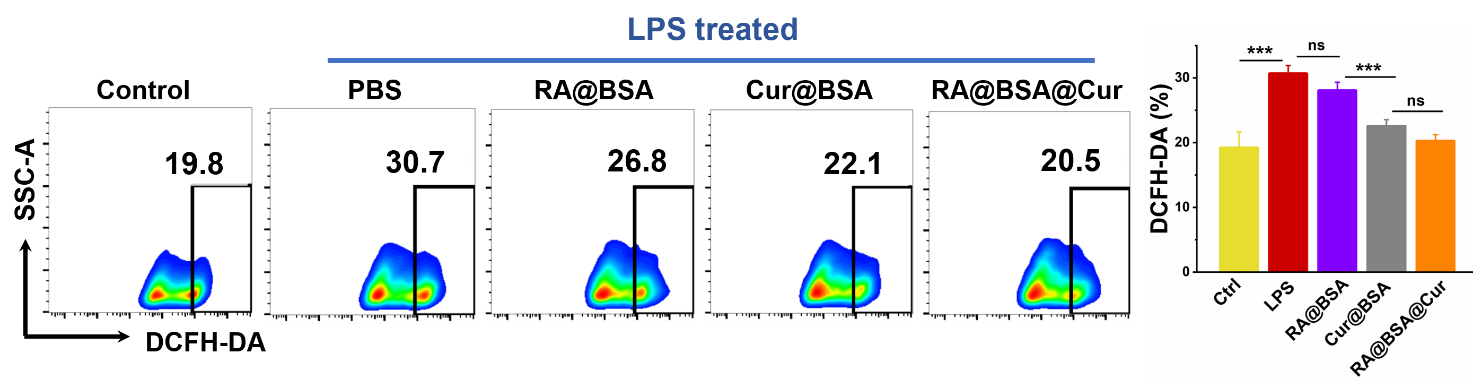


**Figure S6. The change of ROS generation in microglial cell** **treated with RA@BSA@Cur NPs**


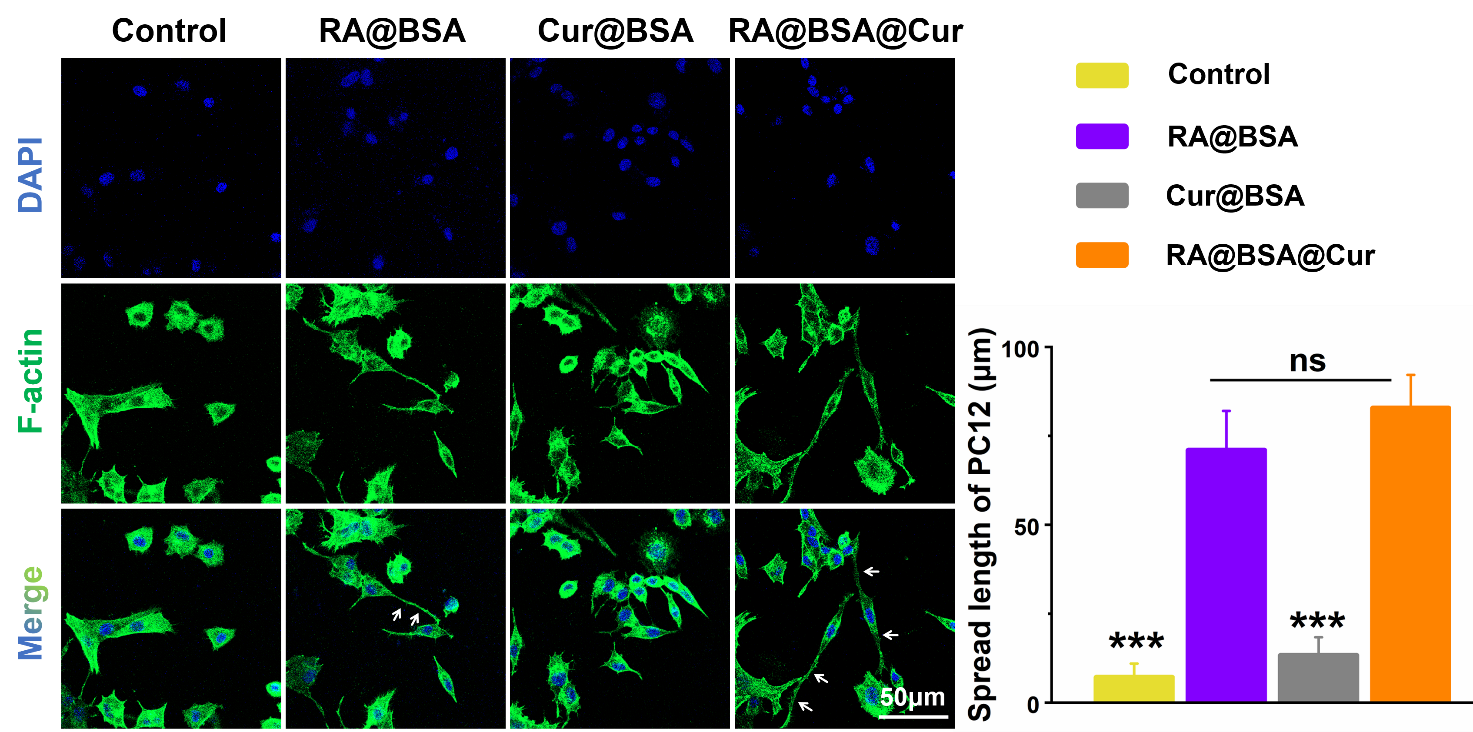


**Figure S7. RA@BSA@Cur NPs promoted the PC12 cell neurite outgrowth.** The neurite length (white arrow) of PC12 cells cultured with RA@BSA, Cur@BSA, and RA@BSA@Cur NPs in physiological condition. Scale bars, 50 μm. (n=3 independent samples). Statistical differences were determined by using the ANOVA with Bonferroni’s multiple comparison test (*p < 0.05, **p < 0.01, ***p < 0.001, ns: no significant; a.u. means arbitrary unit).


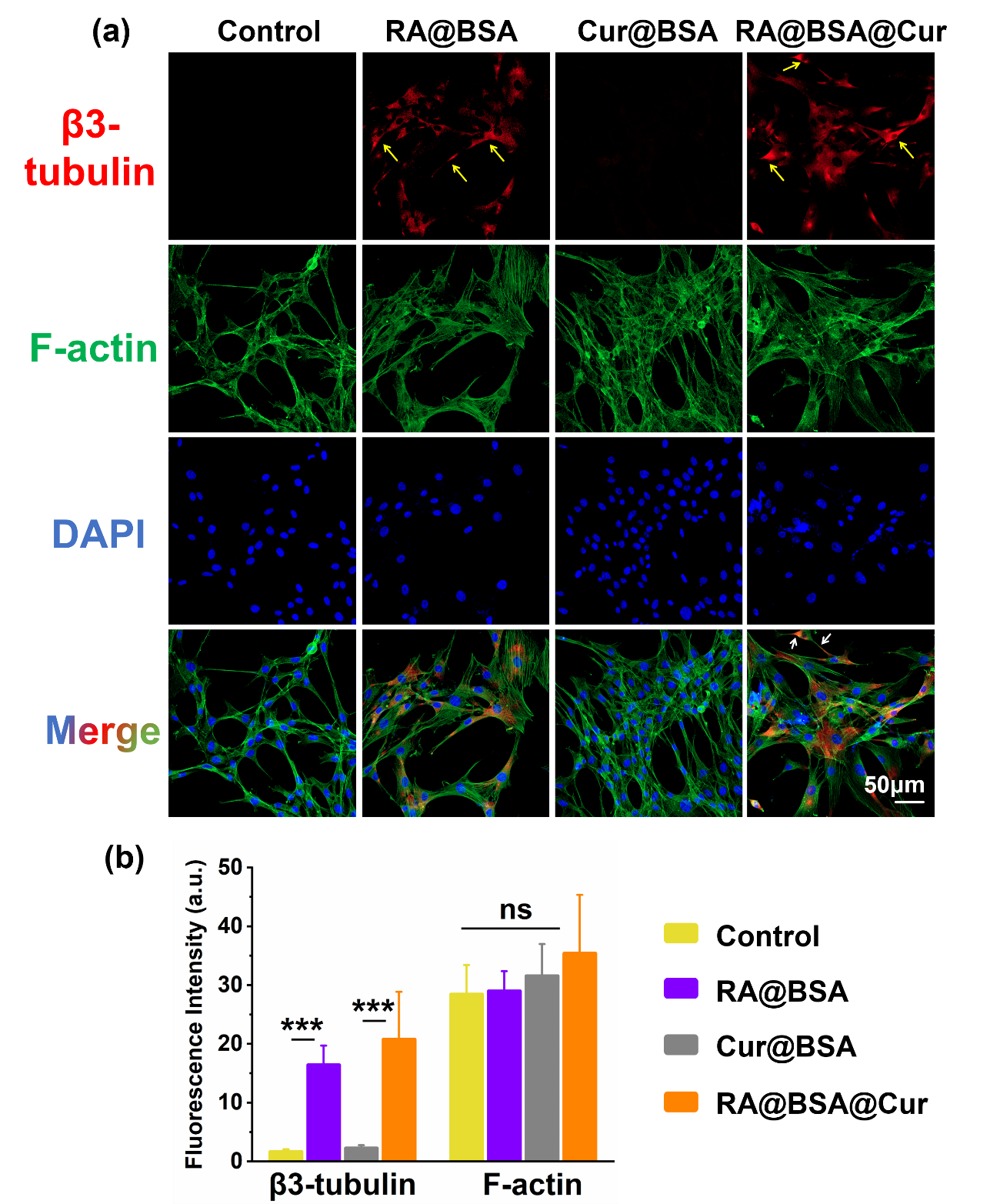


**Figure S8. RA@BSA@Cur NPs on neuronal differentiation of BMSCs.** (**a**) Immunofluorescence-stained BMSCs-derived neural cells (white arrow) for the control, RA@BSA, Cur@BSA and RA@BSA@Cur NPs groups. β3-tubulin (red fluorescence, yellow arrow), F-actin (green fluorescence) and nuclei (blue fluorescence) were stained. Scale bars, 50 μm. (**b**) Quantitative analysis of β3-tubulin and F-actin fluorescence intensity for all groups. (n=3 independent samples). Statistical differences were determined by using the ANOVA with Bonferroni’s multiple comparison test (*p < 0.05, **p < 0.01, ***p < 0.001, ns: no significant; a.u. means arbitrary unit).


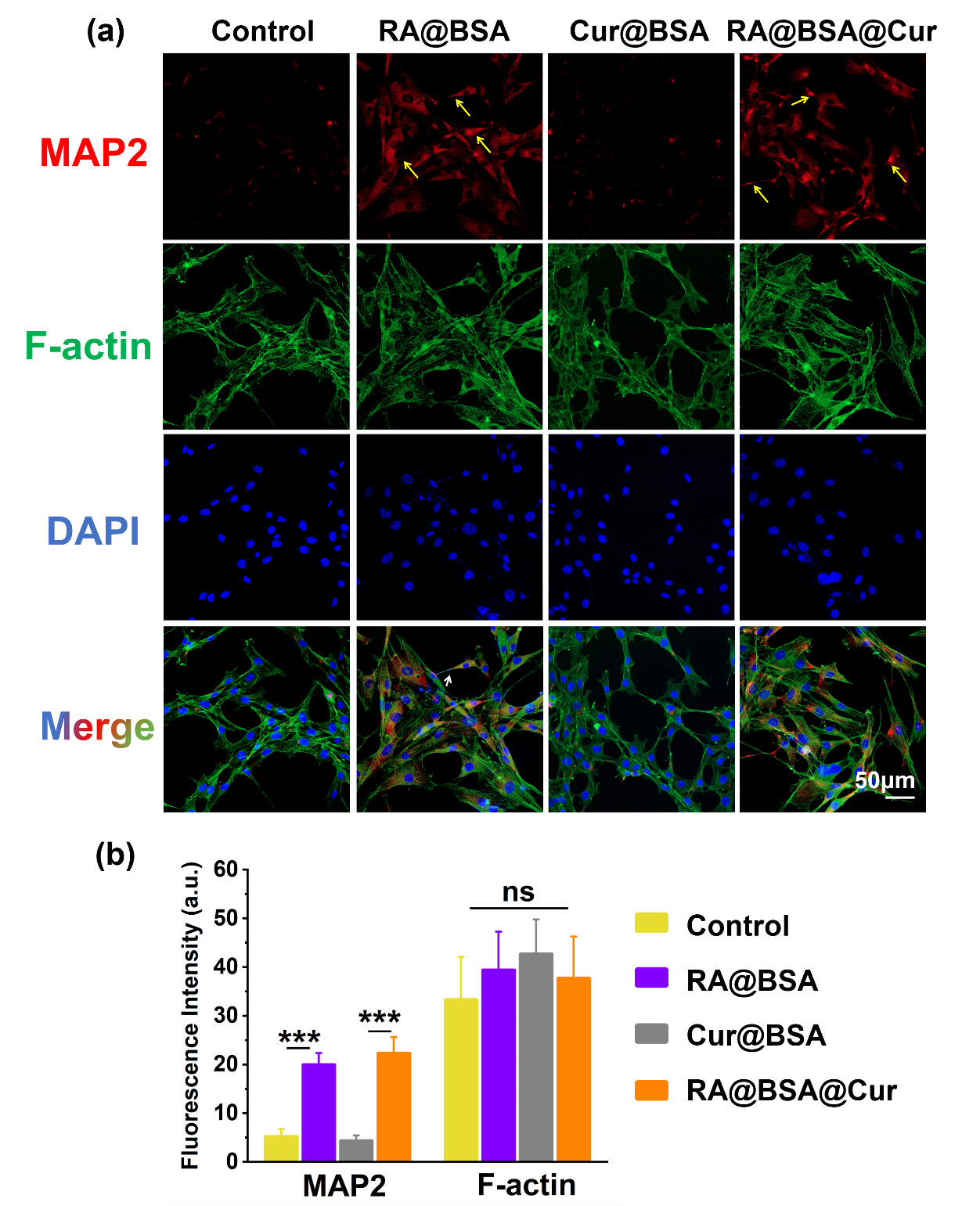


**Figure S9. RA@BSA@Cur NPs on Neuronal differentiation of BMSCs.** (**a**) Immunofluorescence-stained BMSCs-derived neural cells (white arrow) for the control, RA@BSA, Cur@BSA and RA@BSA@Cur NPs groups. MAP2 (red fluorescence, yellow arrow), F-actin (green fluorescence) and nuclei (blue fluorescence) were stained. Scale bars, 50 μm. (**b**) Quantitative analysis of MAP2 and F-actin fluorescence intensity for all groups. (n=3 independent samples). Statistical differences were determined by using the ANOVA with Bonferroni’s multiple comparison test (*p < 0.05, **p < 0.01, ***p < 0.001, ns: no significant; a.u. means arbitrary unit).


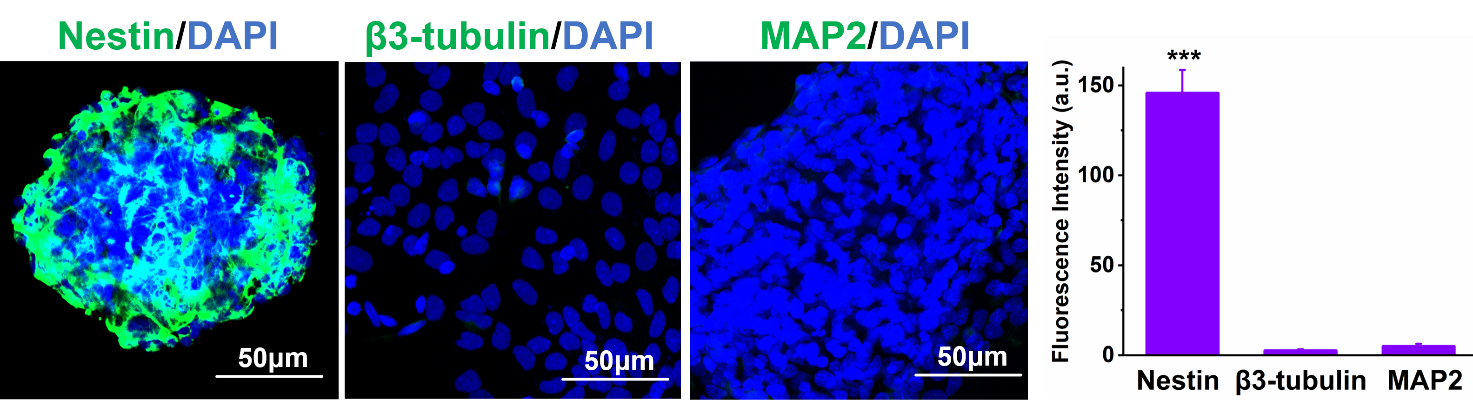


**Figure S10. Primary neural stem cell identified by immunofluorescence stain.** Stem cells were stained with Nestin (neural stem cell marker), β3-tubulin (neuronal marker), and MAP2 (neuronal marker). Scale bars, 50 μm. (n=3 independent samples). Statistical differences were determined by using the ANOVA with Bonferroni’s multiple comparison test (*p < 0.05, **p < 0.01, ***p < 0.001, ns: no significant; a.u. means arbitrary unit).


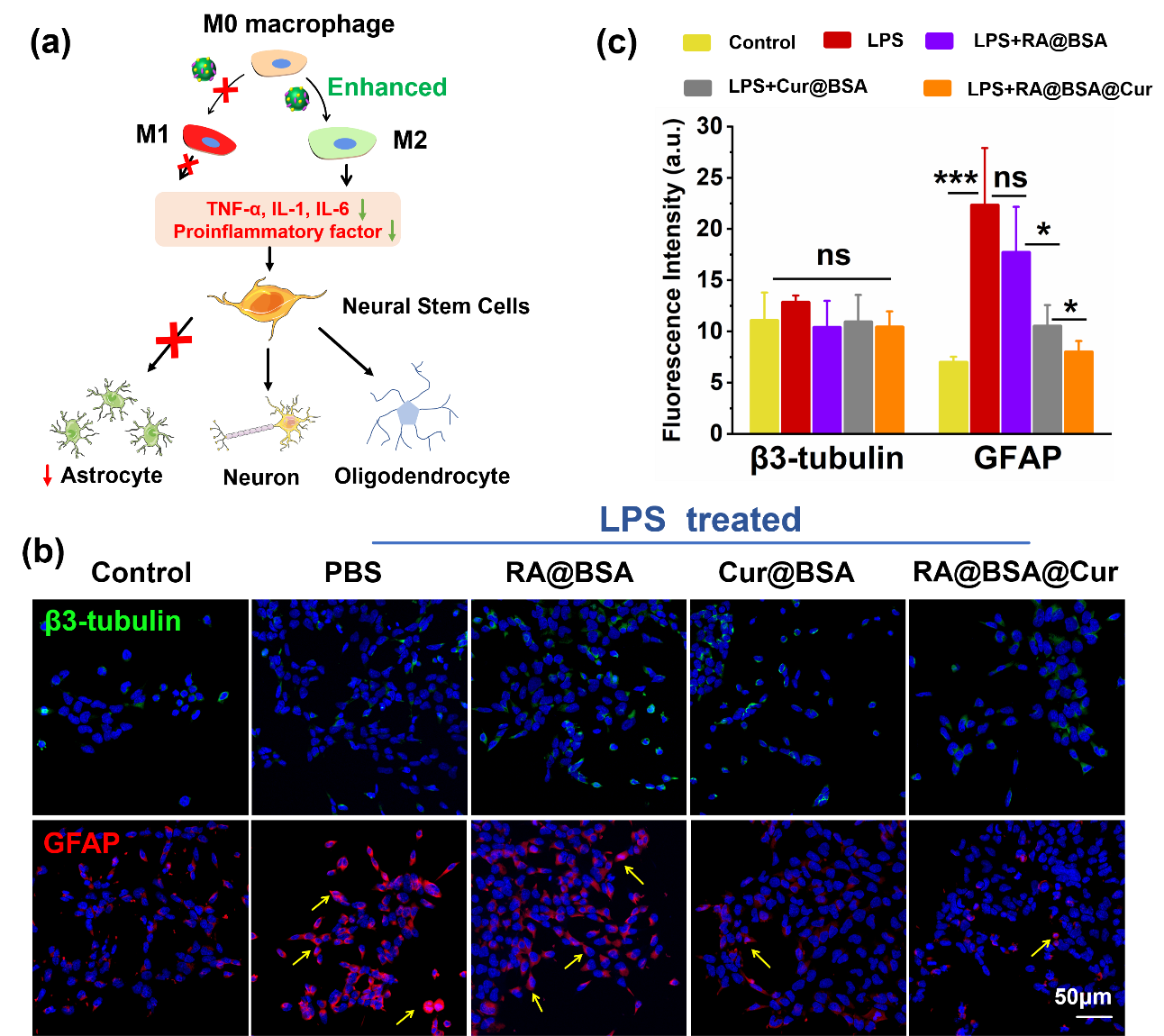


**Figure S11. The RA@BSA@Cur NPs regulated NE4C differentiation through regulating M1 / M2 macrophage polarization.** (**a**) The schematic of RA@BSA@Cur NPs reduced NSC-derived astrocytes through regulating M1 / M2 macrophage polarization. (**b**&**c**) Macrophages treated with different BSA-related NPs in LPS condition for 24 h, the macrophages cell supernatant was collected to induce NE4C differentiation. The immunofluorescence of β3-tubulin^+^ and GFAP^+^ NE4C were observed and quantified. (n=3 independent samples). Neurons (β3-tubulin: green) and astrocytes (GFAP: red, yellow arrow), nuclei (DAPI: blue). Scale bars, 50 μm. Statistical differences were determined by using the ANOVA with Bonferroni’s multiple comparison test (*p < 0.05, **p < 0.01, ***p < 0.001, ns: no significant; a.u. means arbitrary unit).


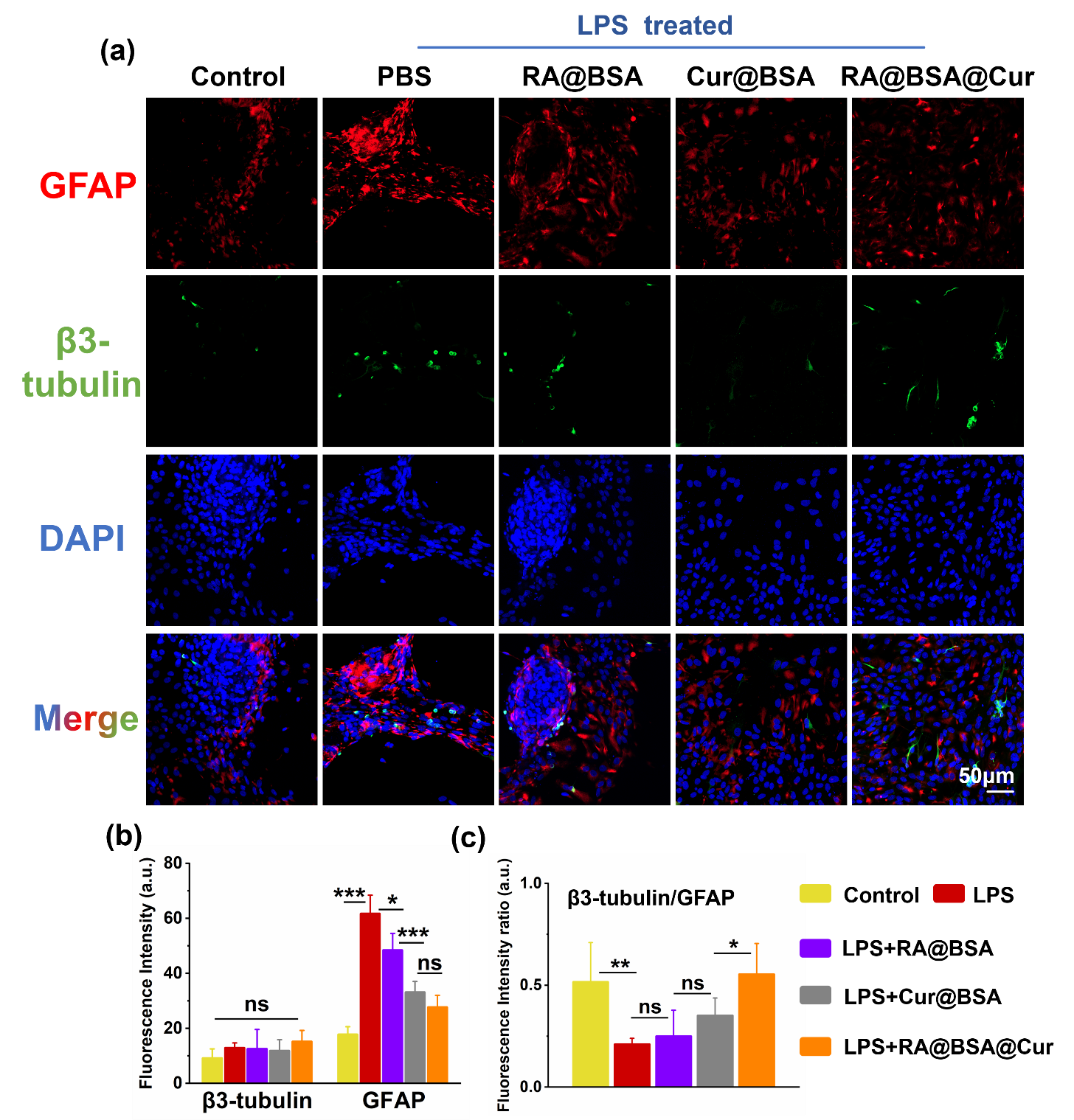


**Figure S12.** **RA@BSA@Cur NPs effect NSC-derived astrocytes production through regulating M1 / M2 macrophage polarization.** (**a**) The macrophages were treated with different BSA-related NPs in LPS condition for 24 h, the cell supernatant of groups was collected to co-culture with primary neural stem cells. The immunofluorescence of neurons / astrocytes in neural stem cell was observed. Neurons (β3-tubulin: green) and astrocytes (GFAP: red), nuclei (DAPI: blue). Scale bars, 50 μm. (**b-c**) quantitative analysis of β3-tubulin / GFAP ratio for all groups. (n=3 independent samples). Statistical differences were determined by using the ANOVA with Bonferroni’s multiple comparison test (*p < 0.05, **p < 0.01, ***p < 0.001, ns: no significant; a.u. means arbitrary unit).


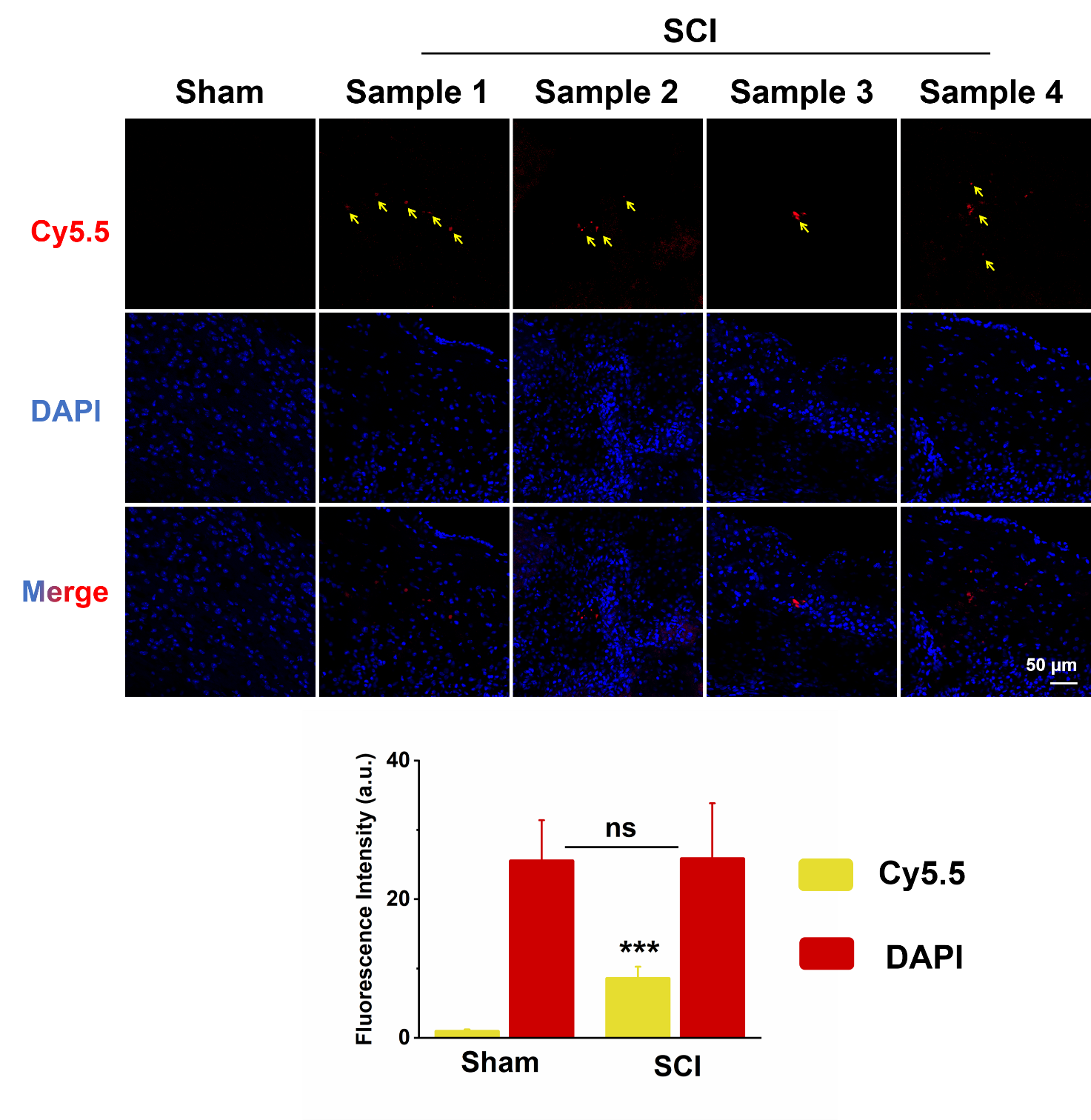


**Figure S13. Microscopic observation of RA@BSA@Cur NPs in the SCI site**. RA@BSA@Cur NPs (red fluorescence, yellow arrow), nuclei (DAPI: blue). Sham: normal spinal cord. Sample 1-4: different areas at spinal cord injury center. Scale bars, 50 μm. (n=3 independent samples). Statistical differences were determined by using the ANOVA with Bonferroni’s multiple comparison test (*p < 0.05, **p < 0.01, ***p < 0.001, ns: no significant; a.u. means arbitrary unit).


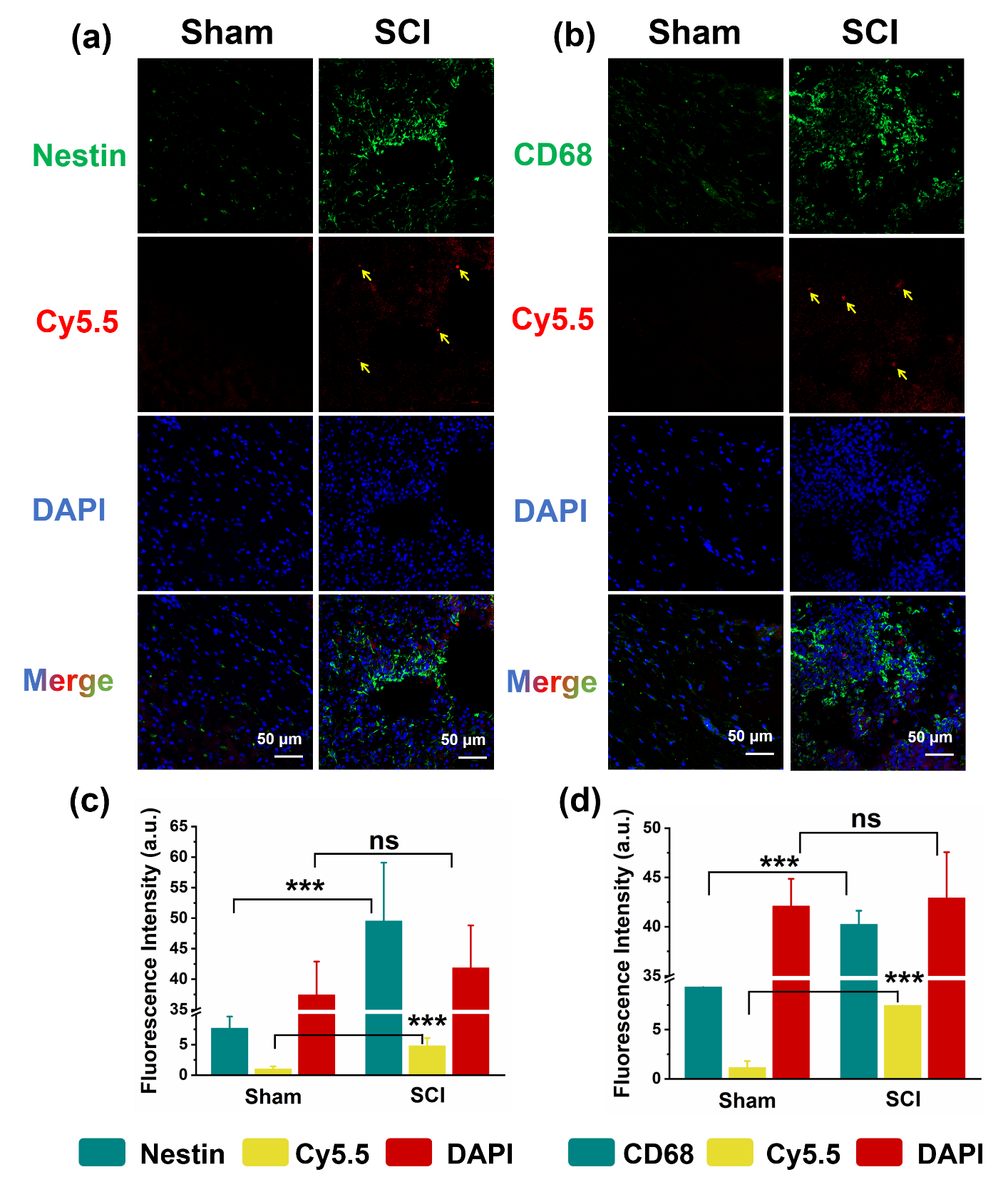


**Figure S14. Microscopic observation of RA@BSA@Cur NPs in the SCI site.** RA@BSA@Cur NPs (red fluorescence, yellow arrow), the neural stem cell (Nestin^+^, green fluorescence, **a**&**c**) and macrophage (CD68^+^, green fluorescence, **b**&**d**). Scale bars, 50 μm. (n=3 independent samples). Statistical differences were determined by using the ANOVA with Bonferroni’s multiple comparison test (*p < 0.05, **p < 0.01, ***p < 0.001, ns: no significant; a.u. means arbitrary unit).


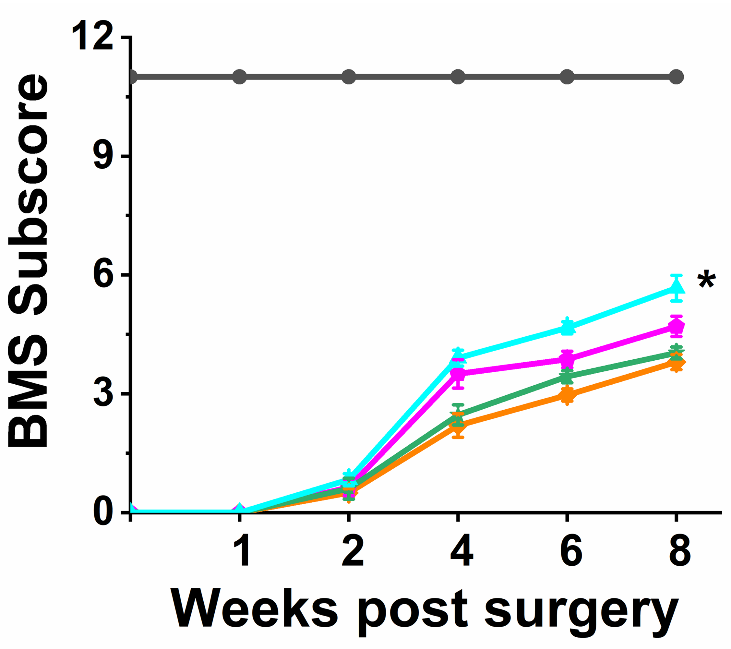


**Figure S15.** BMS Subscore assessment of lower limb fine motor function


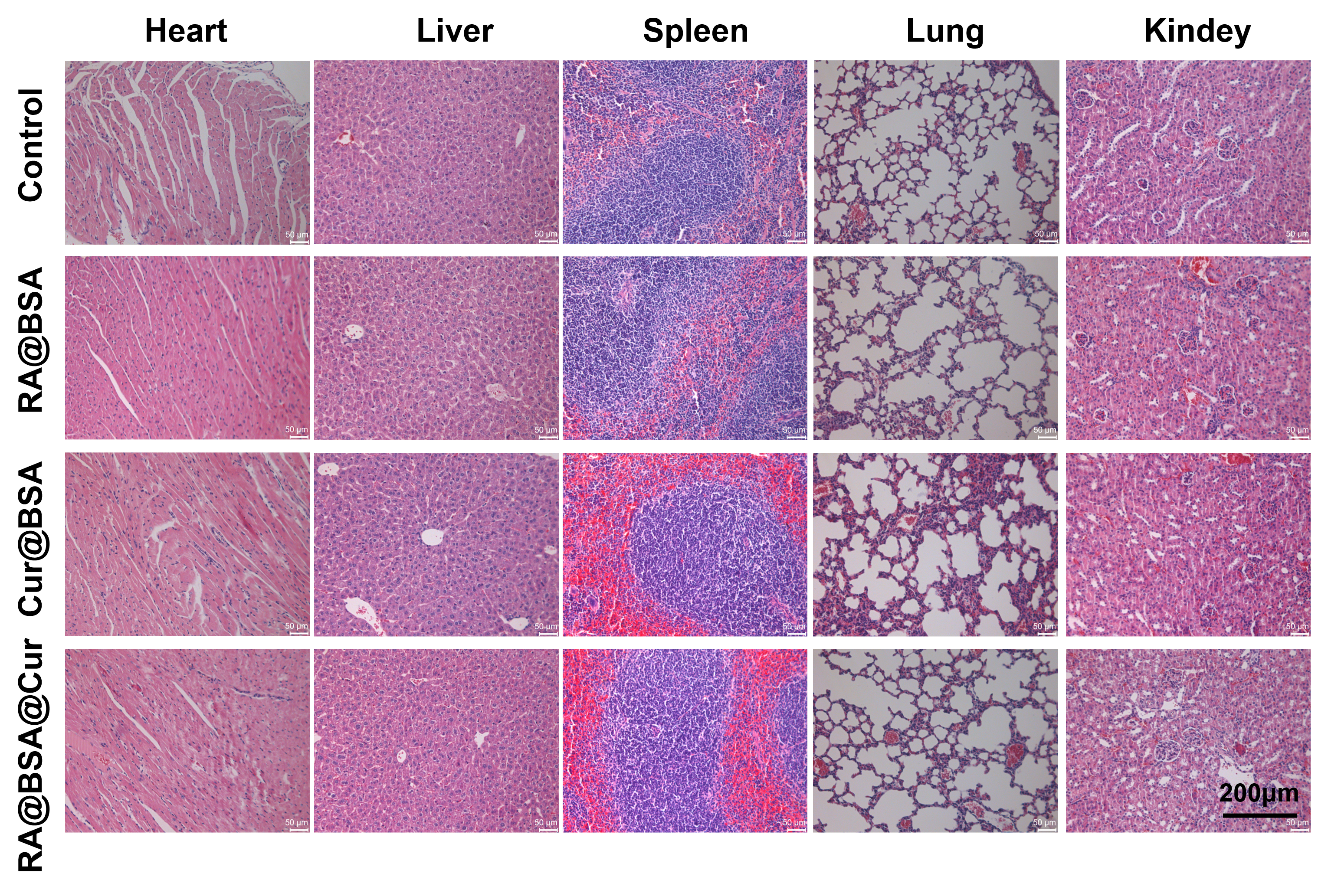


**Figure S16. H&E staining of mice major organs.** The histological changes of mice were determined in heart, liver, spleen, kidney, and lung.
